# The Banana Root Endophytome: Differences between Mother Plants and Suckers and Evaluation of Selected Bacteria to Control *Fusarium oxysporum* f.sp. *cubense*

**DOI:** 10.3390/jof7030194

**Published:** 2021-03-09

**Authors:** Carmen Gómez-Lama Cabanás, Antonio J. Fernández-González, Martina Cardoni, Antonio Valverde-Corredor, Javier López-Cepero, Manuel Fernández-López, Jesús Mercado-Blanco

**Affiliations:** 1 Departamento de Protección de Cultivos, Instituto de Agricultura Sostenible, Consejo Superior de Investigaciones Científicas (CSIC), Campus ‘Alameda del Obispo’ s/n, Avd. Menéndez Pidal s/n, 14004 Córdoba, Spain; cgomezlama@ias.csic.es (C.G.-L.C.); mcardoni@ias.csic.es (M.C.); valverde@ias.csic.es (A.V.-C.); 2 Departamento de Microbiología del Suelo y Sistemas Simbióticos, Estación Experimental del Zaidín, Consejo Superior de Investigaciones Científicas (CSIC), Calle Profesor Albareda, 18008 Granada, Spain; antonio.fernandez@eez.csic.es (A.J.F.-G.); manuel.fernandez@eez.csic.es (M.F.-L.); 3 Departamento Técnico de Coplaca S.C. Organización de Productores de Plátanos, Avd. de Anaga, 11-38001 Santa Cruz de Tenerife, Spain; cepero@coplaca.org

**Keywords:** biocontrol agents, Canary Islands, Cavendish, co-occurrence networks, endophytes, Fusarium wilt, *Musa acuminata*, *Pseudomonas chlororaphis*, *Pseudomonas simiae* PICF7

## Abstract

This study aimed to disentangle the structure, composition, and co-occurrence relationships of the banana (cv. Dwarf Cavendish) root endophytome comparing two phenological plant stages: mother plants and suckers. Moreover, a collection of culturable root endophytes (>1000) was also generated from Canary Islands. In vitro antagonism assays against *Fusarium oxysporum* f.sp. *cubense* (*Foc*) races STR4 and TR4 enabled the identification and characterization of potential biocontrol agents (BCA). Eventually, three of them were selected and evaluated against Fusarium wilt of banana (FWB) together with the well-known BCA *Pseudomonas simiae* PICF7 under controlled conditions. Culturable and non-culturable (high-throughput sequencing) approaches provided concordant information and showed low microbial diversity within the banana root endosphere. *Pseudomonas* appeared as the dominant genus and seemed to play an important role in the banana root endophytic microbiome according to co-occurrence networks. Fungal communities were dominated by the genera *Ophioceras, Cyphellophora, Plecosphaerella,* and *Fusarium*. Overall, significant differences were found between mother plants and suckers, suggesting that the phenological stage determines the recruitment and organization of the endophytic microbiome. While selected native banana endophytes showed clear antagonism against *Foc* strains, their biocontrol performance against FWB did not improve the outcome observed for a non-indigenous reference BCA (strain PICF7).

## 1. Introduction

Banana (*Musa* spp.) is one of the most important fruit and cash crop in terms of production volume and trade in the world [1,2]. The global production of bananas is projected to grow at 1.5% per annum, to reach 135 million tonnes in 2028 [3]. However, a number of soil-borne pests and diseases are principal limiting factors for banana production worldwide [1,4]. Among them, Fusarium wilt of banana (FWB) is considered one of the most destructive diseases affecting this crop (estimated affected area of 100,000 ha and around US $2 billion losses according to Ploetz and co-workers [5]. The causal agent is the soil-borne fungus *Fusarium oxysporum* f. sp. *cubense* (*Foc*) (*Fusarium oxysporum* Schlechtend: Fr. sp. *cubense* (E.F. SM) W.C. Snyder & H.N. Hansen), which infects banana roots, progresses into the xylem of the rhizome, induces wilt, and may eventually kill susceptible cultivars [2,5]. There are four recognized races of *Foc*, which are traditionally identified based on their reaction on different host cultivars [6,7]. Race 1 (R1) isolates attack cultivars Gros Michel (AAA), Silk (AAB), Lady Finger (AAB), Maqueno (Maia Maoli-Popoulu subgroup) (AAB), Pome (AAB), and Pisang Awak (ABB). Race 2 (R2) causes disease in cooking bananas of the Monthan, Bluggoe (ABB) subgroup, other closely related cooking bananas and also affects some bred tetraploids and Enset. Currently, Race 3 is not considered pathogenic in banana, as it only attacks *Heliconia* spp. (tropical American banana relatives) [8,9]. Finally, Race 4 (R4) affects all cultivars belonging to the Cavendish (AAA) subgroup (the most common cultivar (cv.) in commercial banana plantations) and also those susceptible to R1 and R2 [6,7,10,11]. Race 4 is usually split up into subtropical (STR4) and tropical (TR4) races [12]. Whereas STR4 needs predisposing factors to cause FWB (e.g., plants subjected to cold temperatures and other abiotic stresses), TR4, which has been lately identified as a *F. odoratissimum* [13], is able to trigger the disease in Cavendish bananas grown under a broad range of conditions, and it is currently considered the most virulent form of the pathogen [6,7,14,15]. In addition, recent surveys in Asia show that TR4 does not only affect Cavendish bananas but also many other cultivars [16,17]. Nowadays, TR4 has been detected in several banana-producing countries, and its steady and alarming spread has raised a huge concern among producers and in the banana industry [18,19,20]. Thus, research and crop management efforts are currently ongoing to prevent the propagation of the disease, which can seriously compromise the future of this staple food [21,22,23].

Banana is the main intensive agricultural crop in the Canary Islands, which is a Spanish archipelago located in the Atlantic Ocean. Currently, FWB incidence in these islands ranges from 2 to 12%, although occasional disease incidences affecting up to 30% of the plants have been reported in some areas [14,24]. Fortunately, TR4 has not yet been detected in the archipelago, and only attacks by STR4 have been reported [6,23].

The control of FWB is specially challenging because *Foc* is (i) a soil-borne pathogen able to persist in the soil for decades, even in the absence of host plants or within alternate hosts that could remain asymptomatic; (ii) a vascular pathogen that is inaccessible to control measures (e.g., non-endophytic biological control agents (BCA), non-systemic fungicides, etc.) once established within the xylem; and (iii) easily spread by banana vegetative propagation material, soil, workers, machinery, irrigation water, etc. Moreover, the Cavendish banana monoculture expansion favors the dispersion of the disease, which is a particularly concerning scenario when TR4 is present [12,14,23,25]. Thus, an integrated disease management strategy is the best option to mitigate the impact of FWB on susceptible varieties and to increase the durability of available resistant varieties [2,7]. Conventional measures to control FWB include resistant banana varieties, phytosanitary measures, crop rotation, flood-fallowing, application of organic amendments, soil solarization, and fungicides [6].

Within this integrated management strategy, the use of microbial BCA constitutes a promising and environmentally friendly approach [14]. It is accepted that the plant-associated microbiome, usually an important source of BCA, is crucial for the host’s health, development, and productivity [26]. In this framework, the banana belowground microbiome is hypothesized to play essential roles, as it has already been studied in different agricultural pathosystems [27,28], including the banana–*Foc* interaction [1,2,29,30,31,32]. Thus, in-depth analyses of the structure, composition, dynamics, and functionality of the root-associated microbiome are useful to improve disease control approaches. Moreover, a comprehensive knowledge on how the plant-associated microbiome is influenced by factors such as the host genotype and its phenological stages, and/or by environmental and pedological parameters is crucial to understand how the plant holobiont cope with a/biotic stresses. In this sense, it is worth mentioning that root endophytic microbial communities may play essential roles such as, for instance, being an important line of defense against vascular pathogens [33,34]. Hence, the manipulation and harnessing of the endophytic microbiome may help to increase crop production, reduce the incidence of diseases, improve plant resistance, and decrease agrochemicals inputs [2].

Many endophytes are not culturable. For this reason, both culturable-based studies and whole microbial community analysis are required to comprehensively unravel the banana–endophytic community interactions [35,36]. High-throughput sequencing, metabarcoding, and metagenomics sequencing have provided significant advances to understand the behavior of banana endophytes and to identify promising microbes to be used as agro-biotechnological tools in banana crops. Thus, several reports have demonstrated the successful use of different species of culturable endophytic microbes against FWB. For instance, Sekhar and Thomas [37] isolated and identified endophytic bacteria associated with banana cv. Grand Naine, belonging to the Cavendish subgroup, and tested them for antagonistic activity against *Foc.* These bacteria belonged to *Actinobacteria*, *Proteobacteria* and *Firmicutes*, *Pseudomonas aeruginosa* being the most promising species. Comparative analyses between *Foc*-infected and healthy banana plants in Central America identified strains of *Stenotrophomonas* spp. and *Pseudomonas* spp. as potential health indicators that can be used as BCA against FWB [1,29]. Root-associated microbiomes in symptomatic and non-symptomatic banana plants were also investigated with the aim to unravel the responses and functions of the endophytic community during *Foc* infection in Tanzania [31,32]. Proboningrum and co-authors [38] demonstrated that the co-inoculation of two *Bacillus* strains (one rhizospheric and another endophytic) was effective to control FWB and to promote banana growth. Liu and co-workers [2] have recently shown that the banana endosphere bacteriome and mycobiome experienced changes during plant growth and wilting development. Additionally, these authors engineered a banana endophytic enterobacterium to successfully increase FWB resistance and plant growth promotion (PGP).

Suckers are lateral shoots that develop from the mother plants and constitute the main way of banana plants propagation [39]. Whether the sucker microbiome basically originates from the mother plant has not been investigated so far. Considering all the previous background, our study aimed to characterize the structure, composition, and co-occurrence relationships of the banana root endophytome in Canary Islands. We pursued a double-sided goal: (1) to acquire a comprehensive knowledge of the banana root endophytic microbiome by implementing a high-throughput sequencing approach; and (2) to generate a collection of culturable root endophytic bacteria and fungi to be assessed as BCA toward FWB. Thus, we aimed to provide both fundamental knowledge as well as insights into the agro-biotechnological potential of the banana root endosphere microbial communities. The specific objectives were as follows: (1) to unravel and compare, for the first time, the banana root endosphere microbiomes of mother plants and suckers; (2) to understand whether their co-occurrence networks are similar; and (3) to determine a possible correlation between the geographical origin (different islands and different orchards) or physical–chemical soil properties and the endophytic microbiomes. We aimed to test two hypotheses: (i) mother banana plants and suckers share a core microbiome that might play an important role in plant health and development, and (ii) the banana root endosphere harbors beneficial microbes with potential as BCA against FWB.

## 2. Materials and Methods

### 2.1. Banana Roots Sampling and Manipulation

Roots from Fusarium wilt-symptomless banana plants (cv. Pequeña Enana synonymous with Dwarf Cavendish) from eleven farms (Figure S1) representative of different management systems were sampled at three islands (La Palma, Tenerife, and La Gomera) of the Canary Islands archipelago (Table 1). ‘Dwarf Cavendish’ is the main variety cultivated in these islands, representing approximately 81.05% in the period 1995–2019 (Leonardo Amador, I+D responsible of CULTESA-Cultivos y Tecnología Agraria de Tenerife S.A., personal communication) due to its resistance to wind and low temperatures. Two root samples per plant (5 plants per farm) were collected: one from the mother plant (all of them at an unripe stage) and another from the sucker (always reaching 1.50–1.70 m high). In order to ensure that the sampling from mother plants and suckers was carried out unequivocally, root samples (10–15 cm depth) were collected at the opposite sites of the rhizome, and always checking that roots were connected with its corresponding corm (Figure S2). Samples were immediately transferred into polyethylene tubes to avoid excessive desiccation and stored at 4 °C until processing. To prevent contamination by microorganisms attached to the rhizoplane, a thorough root surface sterilization protocol was implemented. Roots were washed with tap water in order to remove adhering soil particles. Surface sterilization was carried out as follows: 96% alcohol for 1 min, sodium hypochlorite (3.7%) for 3 min, and finally 4 rinses in sterile, distilled water. To confirm the effectiveness of the disinfection protocol, aliquots of the final rinse were plated onto Nutrient Agar (NA, Oxoid, Basingstoke, UK) and Potato Dextrose Agar (PDA, Oxoid) plates and incubated at 28 °C for 7 days. After disinfection, roots were air-dried on sterile blotting sheets. Then, subsamples of root tissues from farms F05–F11 (Table 1) were used for the following: (i) DNA extraction and purification aimed to sequence the *16S rRNA* gene and the Internal Transcribed Spacer 2 (ITS2) region for the analysis of the bacterial and fungal root endophytic communities, respectively (see below), and (ii) the isolation of culturable bacterial and fungal endophytes from root tissues (1 g) ground in 5 mL of MgSO_4_·7H_2_O 10mM using sterile mortar and pestle. Banana root samples from farms F01-F04 (all located in Tenerife island) were only meant to isolate culturable endophytes. One of the farms, F09, was sampled in two consecutive years to be used as control in the analysis of whole endophytic communities (Table 1).

### 2.2. Generation of a Collection of Culturable Bacteria and Fungi from the Banana Root Endosphere

Aliquots (100 μL) of 10-fold serial dilutions of the root tissue macerates obtained from each sample were plated under aseptic conditions on five different culturing media, namely NA, NA plus tetracycline (20 µg/mL), PDA, PDA plus lactic acid (0.5 mL/L), and PDA plus streptomycin (50 µg/mL) in order to isolate as many different bacterial and fungal species as possible. For each agar plate evaluated, colonies were selected attending to speed of growth and distinctive morphological characteristics (color, size, shape). Pure cultures originating from single colonies were grown in Luria Bertani agar (LB, Difco, Detroit, MI, USA) for cryopreservation in 2-mL vials containing 33% glycerol at –80 °C until further use.

### 2.3. Physical and Chemical Soil Analysis

Soil from each of the surveyed banana orchards were analyzed to determine their physical and chemical characteristics. The upper soil layer (first 5 cm) was removed, and rhizosphere soil samples were collected (5 to 20-cm depth) following plant roots. At each sampling site (mother plants and suckers), two digs were performed to find and collect the soil firmly adhered to young banana roots. The soil of each dig from the same farm was mixed (1 kg). Soil samples from the farms selected to determine the banana root endophytome (farms F05 to F011, Table 1) were analyzed at the Canarias Explosivos S.A Agricultural Diagnostic Laboratory I + D (Santa Cruz de Tenerife, Spain), using the standardized procedures implemented in this service. Constrained Analysis of Principal Coordinates (CAP or dbRDA) with weighted unifrac distances for bacterial and Bray–Curtis dissimilarities for fungal genera were performed, using the function *ordinate*, to examine the effect of the physical–chemical soil properties on the microbial profiles. Firstly, with the function *capscale*, non-collinear parameters were selected, those with variance inflation factors (VIF) lower than 10. In order to obtain the statistically significant parameters in the resultant distribution, ANOVA tests with the function *anova.cca* were performed. Finally, with the function *cor.test,* the correlation between the significantly different parameters and the genera with ≥ 0.1% relative abundance were computed. Significant correlations (*p*-value < 0.05) with Spearman *rho* ≥ 0.6 were considered strong positive correlations, and those ones with Spearman *rho* ≤ 0.6 were considered strong negative correlations.

### 2.4. DNA Extraction and Illumina Sequencing

Each individual root sample from seven ad hoc selected farms to represent all islands and management systems (i.e., F05–F11, see Table 1) was disrupted by grinding in liquid nitrogen under sterile conditions. DNA from 100 mg of ground root tissues was extracted using the Maxwell RSC (Rapid Sample Concentrator) with the ‘PureFood GMO and Authentication’ Kit (Promega Corporation, Madison, WI, USA) and according to the manufacturer’s instructions. DNA yield and quality were checked both by electrophoresis in 0.8% (*w*/*v*) agarose gels stained with GelRed and visualized under UV light, and by using a Qubit 3.0 fluorometer (Life Technologies, Grand Island, NY). DNA from each individual sample was sequenced using the Illumina MiSeq platform at the genomics service of the Institute of Parasitology and Biomedicine “López Neyra” (CSIC), Granada, Spain. In the first run, prokaryotic libraries were constructed amplifying the hyper-variable V3–V4 regions of the *16S rRNA* gene using the primer pair Pro341F (5′-CCTACGGGNBGCASCAG-3′) and Pro805R (5′-GACTACNVGGGTATCTAATCC-3′) according to Takahashi and co-authors [40]. These amplicons were tagged to be attached to PNA PCR clamps to reduce plastid and mitochondrial DNA amplification [41]. In the second run, eukaryotic libraries were constructed amplifying the ITS2 region using the primer pair ITS4 (5′-TCCTCCGCTTATTGATATGC-3′) [42] and fITS7 (5′-GTGARTCATCGAATCTTTG-3′) [43]. Both runs were sequenced using a paired-end 2x300bp (PE 300) strategy.

### 2.5. Illumina Data Processing

Raw reads were processed with DADA2 [44], which is an open-source package running under R environment. The pipeline tutorials [45,46] were followed for 16S rRNA and for ITS2 amplicons processing. First of all, primers of both datasets were removed with the cutadapt tool [47]. In the filtering and trimming steps of 16S rRNA amplicons, the R1 reads from all the samples in the dataset were trimmed by keeping the first 275 nt, and for the R2 dataset, only the first 250 nt were kept. Then, R1 and R2 reads were merged using default parameters. In the initial Amplicon Sequence Variants (ASV) table obtained (seqtab), reads smaller than 401 nt and larger than 431 were discarded. In the ITS2 amplicons dataset, the length trimming was avoided as recommended in the tutorial. In both datasets, chimeras were detected and discarded with *removeBimeraDenovo* command. Then, the remaining ASV from the 16S rRNA dataset were classified with an 80% bootstrap cutoff to the 16S rRNA reference database of the Ribosomal Database Project (RDP) II, training set v.16 [48], using both the *assignTaxonomy* command from DADA2, and the *classify.seqs* command from MOTHUR v.1.42.1 [49]. The MOTHUR command was used due to the lack of ability of the former command to find mitochondria in the dataset, and the heterogeneity in the taxonomic levels in some genera using this database. These problems are inherent to the RDP database and were corrected in the latest version of MOTHUR’s taxonomy file. Furthermore, ASV identified as mitochondria, chloroplast, and unknown were removed from the dataset. In the case of ASV from the ITS2 dataset, they were classified with the UNITE v.7.2 dynamic database [50]. Finally, ASV accounting for less than 0.015% (bacteria) and 0.017% (fungi) of the total sequences in each dataset were removed according to Bokulich and co-workers [51] and to the mock community used (ZymoBIOMICS Microbial Community Standard II (Log Distribution), ZYMO RESEARCH, CA, USA).

### 2.6. Analysis of Alpha and Beta Diversities

Alpha diversity indices (Observed and Chao1 richness; Shannon and inverse of Simpson diversity (InvSimpson)) were compared with two-way ANOVA for prokaryotes using the R package *car* [52]. For eukaryotes, the Kruskal–Wallis test was used due to the lack of normality/homoscedasticity in these indices, and *p*-values were False Discovery Rate (FDR) corrected by the Benjamini–Hochberg method using the R package *agricolae* [53]. To perform multiple pairwise comparison between the means of groups, Tukey’s Honestly Significant Difference (HSD) test and the Wilcoxon signed-rank test were used for the ANOVA and Kruskal–Wallis results, respectively. Regarding β-diversity, normalization of the filtered ASV sequence counts was performed using the “trimmed means of M” (TMM) method with the BioConductor package *edgeR* [54]. The normalized data were considered to carry out the permutational analysis of variance (PERMANOVA) and permutational analysis of multivariate homogeneity of groups dispersions (BETADISPER), using the functions *adonis* and *betadisper* in the vegan package with 9999 permutations [55]. Weighted Unifrac distances for prokaryotes and Bray–Curtis dissimilarities for eukaryotes were used to compare the variance of β-diversity among and within all treatments/conditions (island, plant phenology, and farm). When applicable, pairwise differences between groups were assessed with the function *pairwise adonis* from the package *pairwiseAdonis* [56]. To visualize the similarities or dissimilarities of the studied communities, those that resulted significantly different from the PERMANOVA analyses were plotted by Non-metric Multi Dimensional Scaling analysis (NMDS) and Principal Coordinates Analysis (PCoA). Ordination analyses were performed using the R package *phyloseq* [57]. Biologically relevant bacterial or fungal phyla and genera were obtained testing for differential taxa relative abundance using proportions in non-normalized counts with the STAMP v.2.1.3 software [58], selecting ANOVA Games–Howell’s post hoc test parameters for multiple groups and Welch’s t-test for two groups comparisons, considering Benjamini–Hochberg FDR for multiple tests correction. Those taxa with statistically significant differences in the two methods previously described were filtered to keep only those in which the difference between proportions was ≥0.5% or the ratio of proportions was ≥2 to be considered biologically relevant and to generate the final selection.

### 2.7. Banana Root Endosphere Core Microbiome Construction

Core bacteriome and mycobiome were built considering only genera that were present in 90% of the replicates of each treatment at minimum according to Hernandez-Agreda and co-authors [59]. Core microbiomes were produced for all the samples and separately for the mother plants and the suckers. Subsequently, core microbiomes were calculated for each island with mother plants and suckers together and also for each island and for each phenological stage. In this last dataset, the cut-off threshold was increased, and only genera present in 100% of the replicates were considered as core members due to each sample group being constituted by a number of replicates (*n* = 5) too low to consider 90% as a cut-off threshold.

### 2.8. Endophytic Banana Root Microbiome Network Construction, Comparison, and Visualization

Microbial (bacterial and fungal) networks were separately constructed for each plant phenological stage (mother plants and suckers). To build these networks, the Molecular Ecological Network Analysis Pipeline (MENAP) website was used [60] following the developer’s instructions [61,62,63,64]. The only parameters changed from default options were the prevalence of the selected ASV (from 50 to 33%), and the correlation coefficient for the similarity matrix construction. Instead of using Pearson’s correlation coefficient, Spearman’s rho using the formula.

(1) $$\mathrm{rs}\;={\;\rho }_{\mathrm{rgx},\mathrm{rgy}}\;=\;\frac{\mathrm{cov}\left({\mathrm{rg}}_{x},{\mathrm{rg}}_{y}\right)}{\sigma_{{\mathrm{rg}}_{x}}\sigma_{{\mathrm{rg}}_{y}}}$$

(where rg_x_, rg_y_ is the rank of the raw score) was selected because it fit better to our dataset profile according to MENAP recommendations. Moreover, 100 random networks were performed to each empirical network to use the standard deviation of the global properties in Student t-test comparisons of the empirical networks between phenology stages in each island. Networks were drawn by using Cytoscape v.3.7.1 [61]. Finally, candidate keystone ASV were plotted in Excel (ZiPi plot) and compared between mother plants and suckers.

### 2.9. In Vitro Antagonism against Fusarium oxysporum f. sp. Cubense

Bacterial and fungal isolates from the banana root endosphere were tested against representative isolates of *Foc* STR4 (isolate CAV-095, VCG 0120, kindly provided by Prof. Altus Viljoen, Stellenbosch University, South Africa) and TR4 (isolate 54006II5, VCG 01213/16, a gift from Prof. Antonio Di Pietro, Córdoba University, Spain). In order to determine the in vitro antagonist activity of banana root endophytes from the collection generated (see Section 2.2), individual drops (10 µL) of each *Foc* strain (containing mycelium and conidia) were plated in the centre of PDA plates. A pre-screening of the entire collection (878 bacterial and 219 fungal strains) was conducted as follows. At four equidistant points from the inoculation spot of the target *Foc* strain, four different endophytic bacteria or fungi isolated from banana roots were inoculated with a sterile loop. Control plates with just a suspension of the pathogen biomass were included in each trial. Then, those bacteria and fungi showing clear antagonist effect were selected, and additional dual cultures were further performed in PDA and NA media plates. Individual drops of *Foc* STR4 and TR4 biomass (i.e., mycelium plus conidia) were plated as mentioned above, and four equidistant 10-μL drops of each preselected microorganism were inoculated. Control plates with just a suspension of *Foc* biomass were included on each experiment. Then, the antagonist activity (i.e., halos or inhibition zones) was scored. The RII was calculated according to the equation: $\left(\mathrm{Rc}-\mathrm{Ra}\right)/\mathrm{Rc}\;$, where Rc is the average radius of the *Foc* colony in the absence of an antagonist microorganism and Ra is the average radius of the *Foc* colony in the presence of an antagonist microorganism (four equidistant points) for nine selected bacteria. These experiments were performed with three biological replicates per each interaction and used media. Mean values were compared by the Welch’s protected Tukey at P = 0.05 using Statistix program (Version 10.0 for Windows. Analytical software 1985-2013). The biocontrol strain *Pseudomonas simiae* PICF7 (formerly *P. fluorescens* PICF7) was included as reference in these assays [65,66].

### 2.10. Molecular Identification of Potential Biocontrol Agents

Banana root endophytes showing the highest RII were molecularly identified. Bacteria eventually selected (95 strains) from the generated collection were identified (at least at genus level) by partial sequencing of the *16S rRNA* and *gyrB* genes. For this aim, isolates were grown in LB medium overnight (28 °C in the dark; 180 rpm). After this, amplifications were performed in a total volume of 25 μL containing 2.5 μL of 10× PCR buffer (50 μM KCl, 10 mM Tris-HCl pH 9 (25 °C), 1% *v*/*v* Triton X-100), 1.5 (*16S rRNA*) or 2 (*gyrB*) mM MgCl_2_, 0.2 μM each primer, 0.2 mM each dNTP, 1.25 U of BioTaq DNA Polymerase (Bioline Ltd., London, UK), and 1 µL of each overnight grown bacterial culture. PCR experiments were performed as previously described by Ruano-Rosa and co-workers [67] except that the initial denaturation step was extended up to 10 min to achieve the bacterial cell lysis and DNA release. 

Molecular identification (at least at the genus level) of the selected fungal isolates (27 strains) was performed by partial sequencing of the Translation Elongation Factor 1-alpha (*tef1*, primers EF1-983F and EF1-2218R) [68] and the Internal Transcribed Spacer region (*ITS*, primers ITS1F and ITS4) [68]. Fungal genomic DNA was extracted using the Hot Sodium HydrOxide and Tris (HotSHOT) method, as described previously [69] with some modifications for the subsequent specific PCRs. The reagents for HotSHOT DNA preparation consisted of an alkaline lysis reagent (A; 25 mM NaOH, 0.2 mM disodium EDTA, pH12) and a neutralizing reagent (B; 40 mM Tris-HCl, pH 4.5). Mycelia samples were collected into a thermal cycler strip tubes. Reagent A (20 µL) was added to the samples and heated to 95 °C for 1 h and rapidly cooled to 4 °C. After that, 20 µL of reagent B were added to each sample. PCRs were performed in 25 μL reaction volumes containing 2.5 μL of 10× PCR buffer, 2.4 mM MgCl_2_, 0.12 μM each primer, 0.2 mM each dNTP, 1.25 U of BioTaq DNA Polymerase and as DNA template, 8 µl of each HotSHOT reaction. PCR assays were carried out following the protocol described by Raja and co-authors [68]. PCR products were purified, sequenced at Sistemas Genómicos S.L. (Paterna, Valencia, Spain), and assembled in ‘contigs’ using the ‘CLC Main Workbench’ (version 7.6.4, CLC Bio, Qiagen, Aarhus, Denmark). The genus (or species when possible) of the strains under study were identified by comparing the resulting contigs with NCBI Blast using the BLASTN algorithm [70].

### 2.11. Phenotyping of Endophytic Bacteria with Biocontrol Potential

In order to identify phenotypes traditionally associated with biological control and/or PGP in banana root endophytes, assays to evaluate phosphatase, catalase, β-glucosidase, protease, amylase, and phytase activities, as well as HCN, 2,3-butanediol (MRVP medium, used according to the instructions provided by Micro Media, Nebotrade Ltd.; Budapest, Hungary) and siderophores production were carried out as previously reported [65,71] and references therein.

### 2.12. Assessment of Biocontrol Effectiveness against Fusarium oxysporum f. sp. Cubense STR4

Based on the results from all phenotyping tests, on the in vitro antagonism assays, and on the molecular characterization, a final selection of three banana root bacterial endophytes (IAS-B-364, IAS-B-793, and IAS-B-944) was made in order to assess their biocontrol performance against FWB. Two independent experiments against *Foc* STR4, isolate CAV-095, were performed under non-gnotobiotic conditions. STR4 was used as a target since it is the most prevalent race in the Canary Islands according to the available information [24,72,73]. The Grand Nain Cavendish variety was selected for biocontrol experiments because it is one of the most commonly used in the banana-producing areas worldwide [74]. In vitro propagated banana plants of this cultivar, kindly provided by CULTESA (Cultivos y Tecnología Agraria de Tenerife S.A), were transplanted into pots (11 × 11 × 12 cm, one plant per pot) each containing an ad hoc prepared substrate made of peat/sand/vermiculite (1:1:1). Pots were randomly distributed in three blocks (15 plants per treatment) in a growth chamber under artificial lighting (14-h photoperiod of fluorescent light at 360 μE/m^2^) and subtropical conditions (26/20 °C day/night, humidity 65%). Prior to bacterial treatments, plants were acclimated for 4 weeks approximately (or until plants developed 4 or 5 true leaves) in the same growth chamber where the experiment was performed. Plants were irrigated as needed and fertilized weekly with 150 mL per pot of Nipofol-K Plus 12-4-36 + microelements (1 g/L) (Fercampo, Málaga, Spain). Bacteria were grown on LB agar plates at 28 °C during 24–48 h. After that, bacterial cells were scraped off from plates with 5 mL of MgSO_4_·7H_2_O 10mM. Then, inocula (1 × 10^8^ cfu/mL in sterile MgSO_4_·7H_2_O 10 mM) for each bacterium were prepared. For each bacterial treatment, the inoculation of banana plants was performed by drenching with 150 mL of a suspension of bacterial cells per pot. Non-bacterized plants (control) were just drenched with 150 mL of sterile MgSO_4_·7H_2_O 10 mM. In both experiments, strain PICF7 was included as a BCA reference. One week after inoculation with the bacteria, plants were challenged with the pathogen by adding 150 mL per pot of a *Foc* STR4 biomass suspension (5 × 10^6^ conidia/mL plus 1.86 mg mycelium/mL). Non-inoculated plants (control) were watered just with 150 mL of tap water. Disease development was assessed weekly and during 90 days after pathogen inoculation by scoring the severity of symptoms on a 0–4 scale according to the percentage of affected leaves and pseudostem (0, no symptoms; 1, 1–33%; 2, 34–66%; 3, 67–100%; 4, dead plant). Analysis of variance (ANOVA) for severity data was carried out. Mean values were compared by the Fisher’s protected Least Significant Difference (LSD) at *p* = 0.05 using Statistix program (Version 10.0 for Windows. Analytical software 1985–2013).

### 2.13. Sequence Accession Numbers

The datasets generated and analyzed during the current study are available in the National Center for Biotechnology Information (NCBI) Sequence Read Archive under the BioProject number PRJNA575333. The *16SrRNA* and *gyrB* genes sequence data of selected culturable endophytic bacteria are available under accession numbers MT445188-MT445196 and MT465269-MT465277, respectively.

## 3. Results

### 3.1. General Characteristics of Sequencing Datasets

A total of 4,486,020 (bacterial) and 7,392,194 (fungal) raw reads were obtained by high-throughput sequencing. Only 3,314,077 (bacterial) and 5,268,838 (fungal) good quality reads were finally retained after the clustering. To avoid an overestimation of the diversity, ASV with less than 0.015% and 0.017% of the high-quality bacterial and fungal reads, respectively, were discarded. A total of 670 bacterial and 248 fungal ASV were eventually considered. For α-diversity comparison, rarefaction was separately performed to the smallest sample of each domain (bacteria and fungi). Finally, 68 samples for bacteria and 65 for fungi, out of 80 total samples for each domain, were retained for downstream analyses (with a Good’s coverage > 99%).

### 3.2. Alpha and Beta Diversity

Comparing richness (observed ASV) and Shannon α-diversity index, a few significant differences were found for both domains (Table 2). The bacterial communities showed significant differences only when comparing mother plants and suckers (Plants comparison in Table 2), particularly in Tenerife and La Gomera islands (Table 2, Figure S3). Meanwhile, the Kruskal–Wallis test for fungal communities showed significant differences only among farms (see Farms comparison in Table 2). Specifically, significant differences (Wilcoxon signed rank test *p*-value < 0.05) were found between farms F07 and F08 in La Palma island; between farms F10 and F11, and F6 and F10 for the mother plants fungal community; between farms F10 and F11 for the mother plants in La Gomera island; and between farms F05 and F06, and F06 and F09 for the mother plants in Tenerife island (Table 2, Figure S4). Data collected from farm F09, sampled in two consecutive years to check consistency of the sampling scheme, did not show significant differences for bacterial nor for fungal communities. 

Concerning β-diversity, bacterial communities showed few relevant differences only when comparing farms. Indeed, the only significant differences were found for Tenerife island communities among farms F05, F06, and F09 (see Farms comparison in Table 3), and for the bacterial communities of mother plants, (i.e., farms F05, F06, F07, F09, F10, and F11; see Farms comparison in Table 3). Meanwhile, fungal communities showed more significant differences at farm, island, and phenological stage levels than those found for the bacterial microbiome. Comparing farms, significant differences were found for La Gomera (between farms F10 and F11) and Tenerife (among farms F05, F06, and F09) islands (see Farms comparison in Table 3). Similarly to bacterial communities, the fungal communities of mother plants showed significant differences when comparing farms (i.e., F05 and F11; F07 and F10). Significant differences were also found in communities of mother plants of Tenerife and La Palma islands (see Farms comparison in Table 3). Moreover, significant differences between farms F10 and F11 were also found for the fungal communities of suckers in La Gomera island (see Farms comparison in Table 3). Comparing mother plants and suckers, significant differences were found only in La Gomera island (see Plants comparison in Table 3). When comparing islands, significant differences were found both for mother plants (between Tenerife and the other two islands) and fungal communities of suckers (among the three islands) (see Plants comparison in Table 3). At the compositional level, the comparison between control data collected in two consecutive years from farm F09 showed significant differences among fungal communities of suckers, but no differences were found among mother plants (see the Farms comparison in Table 3).

### 3.3. Differences in Taxonomical Profile of the Banana Root Endosphere among Islands and between Mother Plants and Suckers

**Figure 1:**
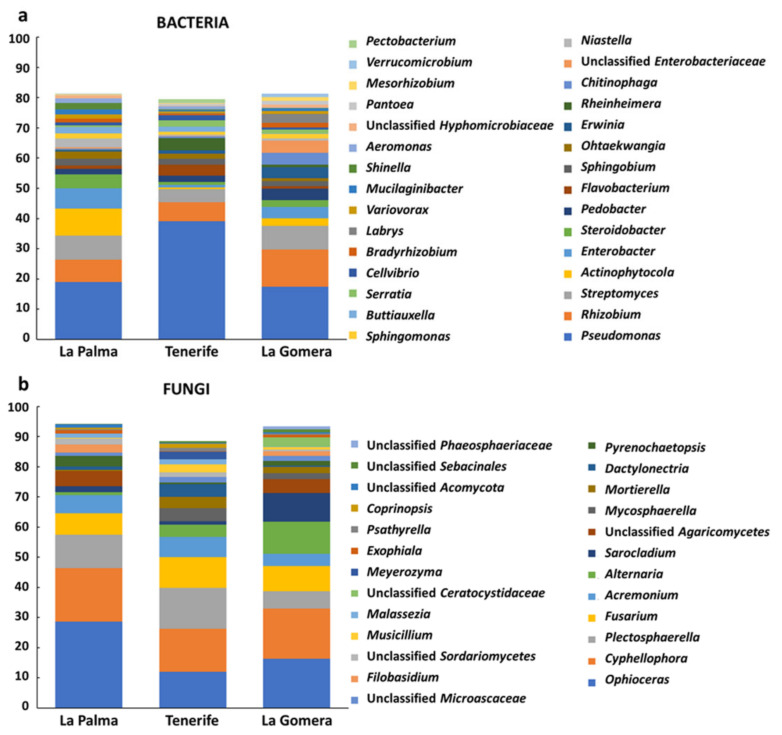
Bacterial (**a**) and fungal (**b**) taxonomical profiles (genus level) of the banana (cv. Dwarf Cavendish) root endosphere communities at the three Canary Islands surveyed in this study. Only genera representing either more than 1% of all sequences for the three islands or for one of them are shown.

When bacterial communities of the banana root endosphere at La Palma, Tenerife, and La Gomera were analyzed 10 different phyla, 19 classes, 35 orders, 70 families, and 157 genera were observed (Table S1). No significant differences were found at the phylum level among the samples analyzed, *Proteobacteria*, *Bacteroidetes*, *Actinobacteria,* and *Firmicutes* (72.3, 12.9, 12.1, and 1.3% of the sequences, respectively) being the most abundant phyla. Bacterial genera *Pseudomonas*, *Rhizobium*, *Streptomyces,* and *Actinophytocola* (25.1, 8.6, 6.7, and 4% of the sequences, respectively) showed the highest relative abundance in the banana root endosphere. Bacterial taxonomical profiles at the genus level (representing either more than 1% of all sequences for the three islands or for one of them) for each of the surveyed islands in this study are shown in Figure 1a. Significant differences in relative abundances were found among farms for *Glycomyces, Kribbella, Labrys,* and *Psychrobacillus* genera. Interestingly, when comparing mother plants and suckers, significant differences were observed at the family level for *Sinobacteracea* (*p*-value = 0.049) that showed a higher abundance in mother plants. In addition, comparison between mother plants and suckers showed significant differences in nine bacterial genera (*Pirellula, Sphingomonas, Beijerinckia, Dongia, Bradyrhizobium, Steroidobacter, Rhizobacter, Reichenbachiella,* and *Ohtaekwangia*, Figure S5). Meanwhile, the most predominant genus *Pseudomonas* showed higher, not significant though, relative abundance in suckers than in mother plants (32.43 vs. 22.41%). In contrast, the relative abundance of genera *Rhizobium*, *Streptomyces*, and *Actinophytocola* were higher in mother plants than in suckers, but these differences were not significant either. The comparison between the bacterial communities of mother plants and suckers also showed significant differences (*p*-value < 0.05) for each island. In La Palma island, four genera (*Beijerinkia, Oxalicebacterium, Rhizobium*, and *Sphingomonas*, Figure S6) were found significantly different, two genera in Tenerife (*Niastella* and *Pseudorhodoferax*, Figure S7) and seven in La Gomera (*Dongia*, *Steroidobacter*, *Labrys, Pseudolabrys, Niastella, Rhodoplanes*, and *Sphingomonas*, Figure S8). However, most of these genera showed relative abundances lower than 2% of the sequences. Other significant differences were detected when comparing mother plants among farms (genus *Labrys,* more abundant in farm F11 followed by farm F10, both belonging to La Gomera Island, *p*-value = 6.91 × 10^−3^) and islands (family *Pseudomonadaceae*, more abundant in Tenerife compared with La Palma and La Gomera, *p*-value = 0.048).

Regarding the fungal communities present in the banana root endosphere of the Canary Islands, 13 phyla, 13 classes, 28 orders, 47 families, and 65 genera were detected (Table S2). The top fungal phylum of banana root endosphere was *Ascomycota* (89%), which was by far followed by phyla *Basidiomycota* (8.9%) and *Mortierellomycota* (2.1%). The same phyla distribution was observed in mother and sucker plants. The phylum *Mortierellomycota* showed significant differences when mother plants were compared both among the three islands (*p*-value 0.045) and among the seven farms (*p*-value = 0.026). In addition, the phylum *Basidiomycota* was significantly different (*p*-value = 0.028) among mother plants from different farms. The fungal community was dominated by the genera *Ophioceras*, *Cyphellophora*, *Plecosphaerella,* and *Fusarium* (19, 16.2, 10, and 8.6% of the sequences, respectively). Fungal taxonomical profiles of these genera, and others representing either more than 1% of all sequences for the three islands or for one of them, are shown in Figure 1b. The second most abundant fungal genus (*Cyphellophora*) showed statistically significant differences in its relative abundance between mother plants and suckers (*p*-value 0.016), being more abundant in mother plants (22 vs. 8% of the sequences, respectively) (Figure S9). Likewise, differences between mother plants and suckers were found in La Gomera at the order level, since the relative abundance of *Chaetothyriales* was significantly higher in mother plants (*p*-value 0.011). Other significant differences in fungal relative abundances were found when suckers of the three islands were compared. For instance, significant differences were observed for the genus *Sarocladium* (*p*-value = 0.018), which was more relatively abundant in La Gomera (Figure S10).

### 3.4. Defining the ‘Dwarf Cavendish’ Root Endosphere Core Microbiome

**Figure 2:**
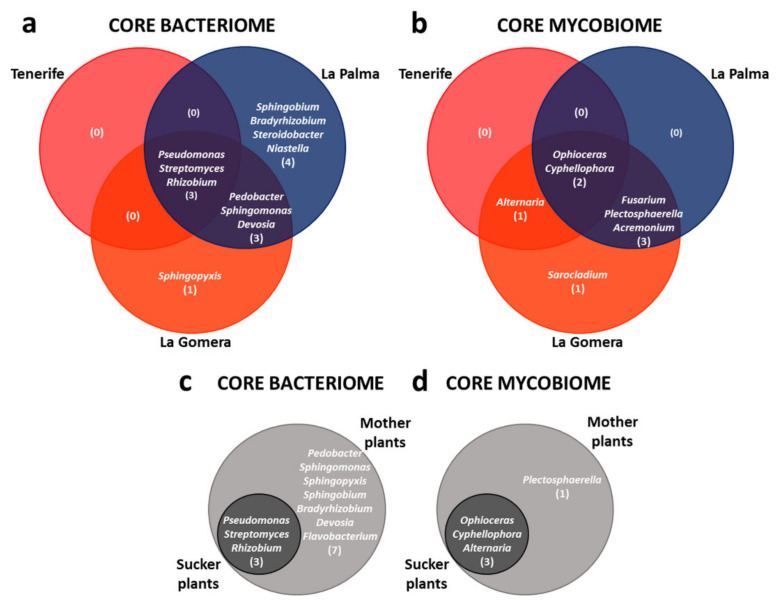
(**a**) Bacteriome and (**b**) mycobiome cores of the banana root endosphere (cv. Dwarf Cavendish) at Canary Islands (La Palma, Tenerife, and La Gomera); (**c**) bacteriome and (**d**) mycobiome cores shared by mother plants and suckers.

Regarding bacteria, more than 96% of the sequences (accounting for the average relative abundance of all samples) were classified at the genus level, accounting for 157 genera. Remarkably, the ‘Dwarf Cavendish’ root endosphere core bacteriome of the three surveyed islands was composed of a very low number (3) of genera, representing 41% of the total sequences and distributed just among *Pseudomonas* (27%), *Rhizobium* (8%), and *Streptomyces* (6%) (Figure 2a and Table S3). 

Concerning the fungal community, around 90% of the sequences (accounting for the average relative abundance of all samples) were classified at the genus level, representing 64 different genera. Similar to the core bacteriome, the core mycobiome of the ‘Dwarf Cavendish’ root endosphere at Canary Islands was formed only by a very limited number of genera, namely *Ophioceras* (representing 18% of the total sequences) and *Cyphellophora* (15%) (Figure 2b and Table S4).

Clear differences at the genus level for individual core microbiomes among the three surveyed islands were observed. Individual core bacteriomes of ‘Dwarf Cavendish’ plants at Tenerife, La Gomera, and La Palma islands were made up of 3, 7, and 10 bacteria genera, respectively (Figure 2a). Hence, at the bacterial level, La Palma showed the largest core bacteriome out of the three banana-producing regions examined. With regard to the mycobiomes, the La Gomera core showed the largest number of genera (7), followed by La Palma (5) and Tenerife once again with the smallest number of genera (3) (Figure 2b).

Likewise, differences at the genus level for mother plants and the suckers core microbiomes were observed. The core bacteriome shared by banana mother plants and suckers was formed by the same three bacterial genera identified for the Canary Islands core bacteriome. The number of sequences found for these genera represents more than 38% of the total sequences (Table S3). The core bacteriome of mother plants consisted of a much higher number of genera than that observed in suckers (10 vs. 3, see Figure 2c). However, with respect to the fungal community, the core mycobiomes of mother plants and suckers were rather similar. The mycobiome of mother plants consisted of four genera, while that of the suckers was formed by just three (Figure 2d). Interestingly, both the core bacteriome and the core mycobiome of suckers were smaller than the cores of the mother plants, and they constituted a subset of the latter (Figure 2c,d). 

### 3.5. The Phenological Stage Influences the Co-Occurrence Networks Topology of the Banana Root Endosphere Microbial comMunities

**Figure 3:**
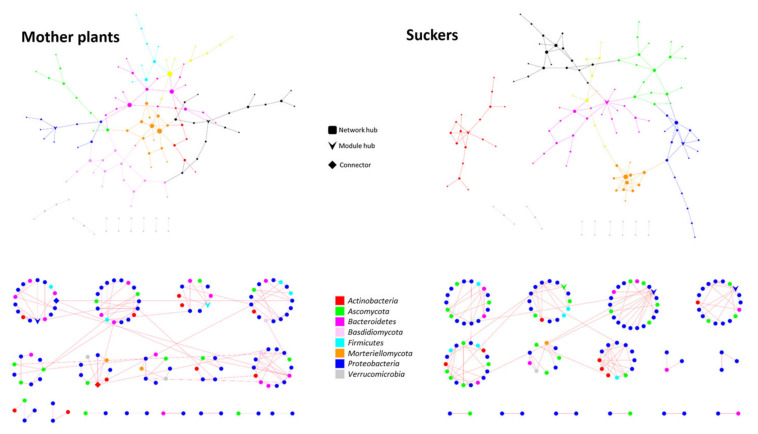
Co-occurrence networks of microbial communities from the banana root endosphere of mother plants (**left**) and suckers (**right**). In the upper panels, each color represents different modules in the networks. Modules with less than five nodes are drawn in gray color. The bottom panels show the modular layout of the networks with nodes colored according to their phylum. Red lines (links) indicate negative interactions.

Microbial networks were separately constructed for mother plants and suckers. Co-occurrence networks analysis revealed that microbial community interactions in banana roots are quite different depending on the plant phenological stage (Table 4). Different network topologies were observed when comparing mother plants and suckers (Figure 3). Indeed, a remarkable result was that the network in the suckers was significantly more complex (higher avgK and avgCC) and showed higher modularity (M) and Geodesic Distance (GD) than that in mother plants (Table 4; Figure 3).

This indicates that microorganisms in the suckers are more compartmentalized than those inhabiting mother plants, as reflected by avgCC values (Table 4), suggesting that individuals from one module within the sucker’s network have a higher degree of interaction than those in a module of the mother plant’s network.

**Figure 4:**
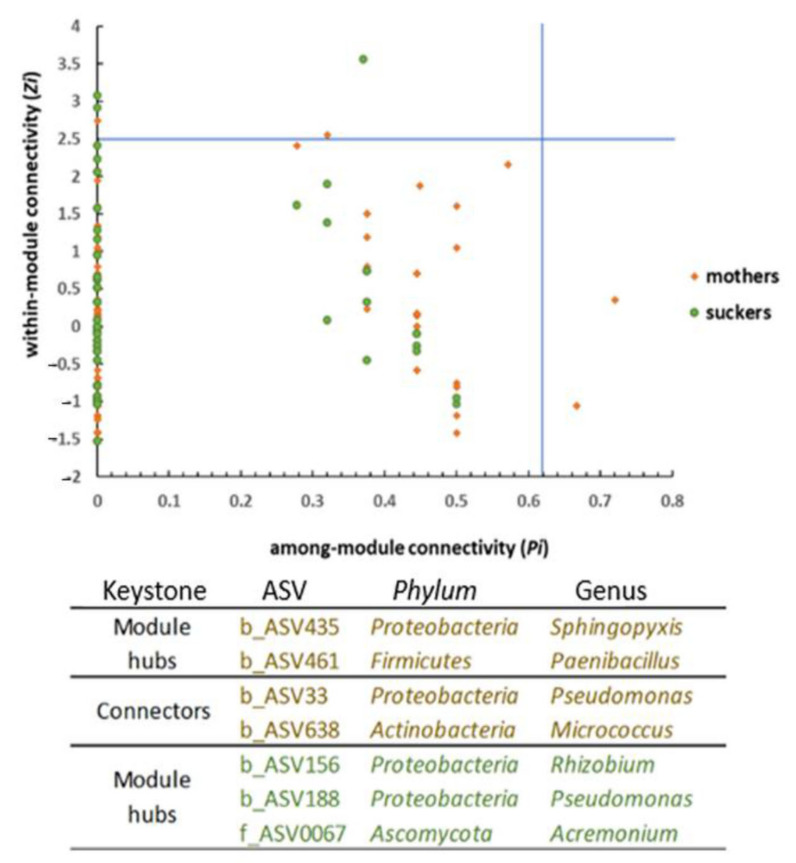
ZiPi plots highlighting the keystone Amplicon Sequence Variants (ASV) of the banana root endosphere microbial network of mother plants and suckers. Module hubs have a Zi value ≥ 2.5 and connectors have a *p* value ≥ 0.62. Details of each keystone ASV are shown in the table.

Furthermore, the network of the suckers showed higher within-module connectivity (Zi) with three module hubs but lower among-module connectivity (Pi) (without connectors in this case). In contrast, the network of mother plants showed two module hubs (Zi) and two connectors (Pi) (Figure 4). On the one hand, *Pseudomonas* was a keystone ASV (b_ASV33) playing a role as a connector in the mother plants’ network. Additional keystone ASV in the mother plants’ network were *Paenibacillus, Sphingopyxis,* and *Micrococcus.* On the other hand, a different *Pseudomonas* ASV (b_ASV188) was also a keystone functioning as a module hub (Figure 4) in the network of the suckers. Moreover, *Rhizobium* (second most abundant genera according to the high-throughput sequencing analysis) and *Acremonium* were also identified as keystone ASV for this latter network. Surprisingly, both networks consisted of a high number of modules among which almost all interactions (links) were negative (Figure 3, red lines). In fact, PEP was lower than 1% in both cases (Table 4). 

### 3.6. Scarce Influence of Soil Properties in the Banana Root Endophytome

**Figure 5:**
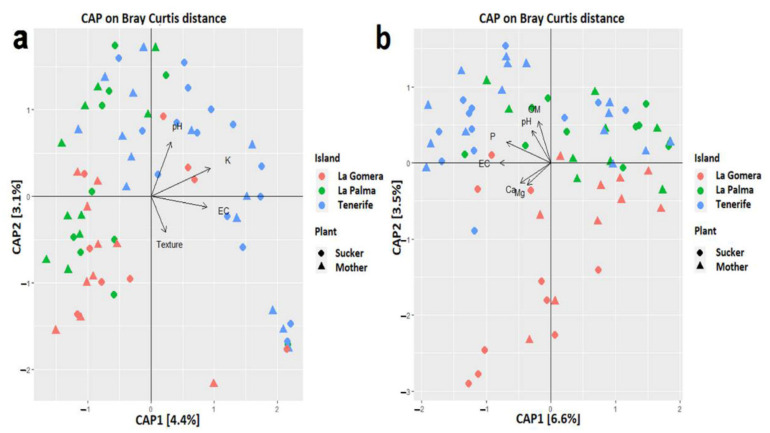
Constrained Analysis of Principal Coordinates (CAP) of (**a**) bacterial communities by treatment (island and phenological stage) on weighted Unifrac distances and (**b**) fungal communities by treatment on Bray–Curtis dissimilarities, both with all the independent physical–chemical parameters. EC, electrical conductivity; OM, organic matter content.

Overall, the physical–chemical soil parameters of the banana farms here surveyed (Table S5) did not contribute significantly to the microbial profiles. Although some parameters differed among farms (for example, pH ranged from 5.4 to 8, organic matter from 3.4 to 9.7%, electrical conductivity from 2.44 to 17.68 mS/cm, and some cations differed by an order of magnitude, Table S5), none of them proved to be a good predictor of the composition of the banana root endophytome. Indeed, the statistically significant parameters (Figure 5) explained less than 10% of the samples distribution in the CAP plot, indicating the low weight of these parameters. Furthermore, no bacterial genus was significantly correlated to any of these parameters (*rho* > 0.6 or <−0.6), and only one fungal genus, *Pyrenochaetopsis* (*p*-value = 1.92 × 10^−8^), was negatively correlated (*rho* −0.62) to electrical conductivity. 

### 3.7. The Banana Root Endosphere Is a Reservoir of Plant Growth Promoters and Antagonists against Fusarium oxysporum f.sp. cubense

A collection of 1097 single/pure (80% bacteria and 20% fungi) isolates was obtained from the root endosphere of ‘Dwarf Cavendish’ plants grown in the Canary Islands (Table 1). Based on the results from in vitro antagonism assays against representative isolates of *Foc* STR4 and TR4 (see the Experimental Procedures section), 122 isolates were eventually preselected for further analysis. The presence of phenotypes traditionally associated with biocontrol and/or PGP was assessed in this subset of banana root endophytes. Table 5 summarizes the number and percentage of isolates showing positive results for the activities tested. 

### 3.8. Prevalence of Culturable Pseudomonas spp. in the Root Endosphere of ‘Dwarf Cavendish’ Grown in Canary Islands

The 122 preselected isolates showing antagonism against *Foc* were molecularly identified, when possible, at the genus level (Table S6). Data showed a low number of bacterial genera in this subset of banana root endophytes, which is in agreement with results from the high-throughput sequencing approach implemented in this study (see above). *Proteobacteria* was the dominant phylum to which most of the preselected culturable banana root endophytes with antagonistic activity were assigned, and more specifically representatives of the *γ-Proteobacteria* class. Similarly to the results obtained from high-throughput sequencing (Figure 1a), the prevalent bacterial genus was *Pseudomonas* (68% of the molecularly identified culturable bacteria of the preselected subset), including mainly representatives of the subspecies *piscium*, *aurantiaca,* and *aureofaciens* of *Pseudomonas chlrororaphis*. Other culturable bacterial endophytes showing antagonist activity against *Foc* were *Pantoea* sp., *Rhizobium* sp., *Flavobacterium* sp., and *Luteibacter* sp. Fungal endophytes were less abundant than bacteria, which is also in good agreement with results obtained from high-throughput sequencing. Thus, representatives of genera *Gloeotinia, Fusarium, Plectosphaerella, Gliocladium, Epiccocum*, *Acremonium,* and *Alternaria* were isolated and identified (Table S6). Interestingly, massive sequencing also showed that *Fusarium* and *Alternaria* were two of the most predominant genera in the banana root endosphere (Figure 1b). Potential human (e.g., *Serratia marcescens*, *Mycobacterium peregrinum*, *Chrysobacterium indolegens, Alternaria alternata*), plant (e.g., *Alternaria alternata, Erwinia* spp., *Fusarium oxysporum* f.sp. *dianthi, F. proliferatum, Plectosphaerella cucumerina*), and insect (e.g., *Verticillium insectorum* and *Pochonia clamidosporia*) pathogens were identified as well. These endophytes were discarded from further characterization because of their null applicability in agro-biotechnology.

### 3.9. Biocontrol Performance of Selected Banana Root Endophytes against Fusarium Wilt

**Figure 6:**
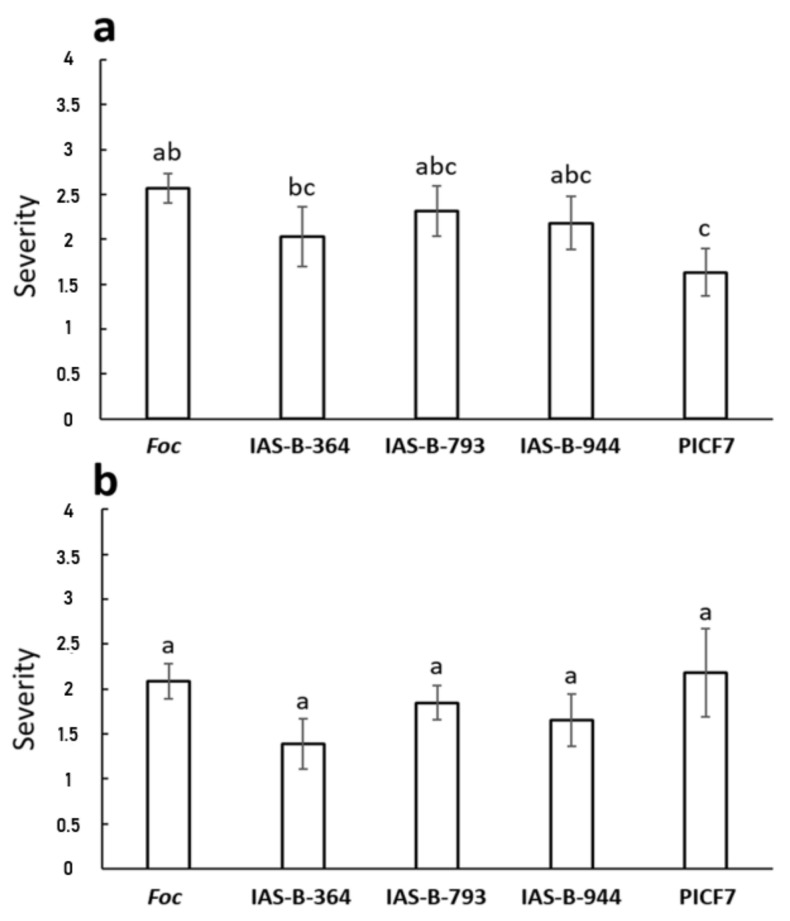
Biocontrol performance of selected banana root endophytic *Pseudomonas* spp. strains against *Fusarium oxysporum* f. sp. *cubense* STR4 (*Foc*). Results shown in panels (**a**,**b**) correspond to two independent experiments. *Pseudomonas simiae* PICF7 was used as biocontrol reference strain. Symptoms severity scale: 0, no symptoms; 1, 1–33% affected foliage; 2, 34–66%; 3, 67–100%; 4, dead plant. Error bars represent the standard error of the means (*n* = 15). Different letters indicate significant differences (LSD test, *p* < 0.05).

Nine bacterial endophytes displaying the best in vitro relative inhibition index (RII) against STR4 and TR4 (Table S7), and the most promising phenotypes related to biocontrol and PGP were eventually selected for further characterization (Table S8 and Table 6). Molecular identification of these bacteria is also shown in Table 6. Remarkably, all of them were molecularly identified as *Pseudomonas* spp. Plants were only challenged with STR4, since this race is prevalent in Canary Islands and TR4 is not present, according to the available information, in the archipelago. Non-inoculated plants (control treatment) and plants just treated with the three *Pseudomonas* spp. strains that were eventually selected for these bioassays showed normal development in the two independent experiments performed. The first FWB symptoms (yellowing of the first true leaves) in *Foc*-inoculated plants appeared at 15–19 days after inoculation with the pathogen. Results from a first experiment only showed a disease reduction trend in plants pretreated with the endophyte IAS-B-364, although the trend was not significant. Only plants treated with the BCA reference strain PICF7 showed a significant (*p* < 0.05) reduction of disease symptoms caused by STR4 compared with the control treatment (Figure 6a). In a second experiment, none of the selected banana root endophytes was able to control FWB development. In this case, strain PICF7 did not control the disease either (Figure 6b).

## 4. Discussion

The banana-associated belowground microbiome from Fusarium wilt symptomatic and asymptomatic plants has previously been studied by culturable and non-culturable methods [1,2,29,31,32,37,75,76]. The present study is the first one to perform an in-depth comparative characterization of the root endophytic microbial communities of banana when plants are in two different phenological stages: mother plants (at fruiting stage, unripe) and suckers (juvenile stage). In addition, possible correlations with soil type, crop management, and geographical origin (farm location) in the Canary Islands were analyzed by implementing high-throughput sequencing and traditional microbiological tools.

One of the main results of our study was that culturable and non-culturable approaches provided concordant information and showed that the banana root endosphere at the Canary Islands harbors microbial communities with rather low diversity. According to high-throughput sequencing results, and at the bacteria phylum level, the banana root endosphere was dominated by *Proteobacteria* (in agreement with the composition of the culturable bacterial collection generated) and *Bacteroidetes*, and to a lesser extent by *Actinobacteria*. This result agrees with that from Kaushal and co-workers [31], who recently studied the microbiome associated with FWB symptomatic and non-symptomatic bananas, and also with results from other authors [75,77,78]. In contrast, another work [37] mostly identified representatives of the phylum *Actinobacteria* among the endophytic bacteria present in banana plants. However, these authors only implemented a culturable-based approach. The most prevalent endophytic bacterial genera inhabiting the Canarian banana root samples here analyzed were *Pseudomonas*, *Rhizobium*, *Streptomyces,* and *Actinophytocola* (Figure 1a). The highest relative abundance of *Pseudomonas* spp. fully correlated with the largest percentage of the molecularly identified culturable bacteria. Interestingly, Kaushal and co-authors [31] also reported *Pseudomonas* as dominant genera in non-symptomatic banana plants surveyed in Tanzania. Likewise, Rossman and co-workers [75] also found an abundance of *Pseudomonas* spp. in the microbial community of bananas, although in their study, the genus *Enterobacter* was the most abundant (fifth most abundant genus in our study, Figure 1a). However, our results do not agree with those obtained by Liu and co-workers [2], who reported *Caulobacter* and *Paracoccus* as the most abundant genera in healthy and wilting plants in China. Regarding the banana root endophytic fungal community, *Ascomycota* was the prevalent phylum found in Canary Islands, which is in agreement with results obtained in two African locations [32].

Concerning the α- and β-diversity, few significant differences were observed among the three studied islands and the surveyed farms. Remarkably, the most significant differences were found when comparing microbial communities, both bacterial and fungal, between mother plants and suckers. Banana plants are reproduced clonally through suckers in the field and in nurseries or by in vitro culturing. According to Kaushal and co-authors [32], since banana is a perennial crop whose successive crop cycles are established from suckers, these would be automatically colonized with the same (or part of the) microbiome present in their mother plants. Therefore, these authors suggested that the banana corm would be the principal source of bacteria and fungi for the suckers. The results obtained in our study revealed that although all taxa constituting the core microbiome of the suckers were present in the core microbiome of the mother plants, not all components of the latter were found in the former. Interestingly, these results are in line with those obtained from co-occurrence networks analysis that showed notable differences between microbial community interactions taking place inside sucker roots and that of the mother plant roots. None of the generated networks were very complex (Figure 3). Even though the number of ASV identified in the mother plants’ network was higher than that of the suckers, the network from the latter showed higher significant parameters. That is, the network of the suckers would be the most complex. It has been suggested that plants with more complex community networks can better cope with environmental changes/stresses, including those caused by the presence of pathogens [34,79,80,81]. Furthermore, the increase in avgCC, M, and GD network parameters, which means more interactions among individuals belonging to the same module but a lower number among individuals from different modules, can be a strategy to protect the microbial community [34,82] from possible disturbances caused at early stages of the suckers’ development. In fact, this type of network topology may help to reduce or prevent the spread of the alteration provoked by a pathogen, containing it in one or few modules [34]. More detailed studies would be needed to confirm whether this network complexity is not conserved when the plant reaches the adult stage. It was striking to observe that nearly all interactions in both networks were negative (Figure 3). According to Faust and co-authors [83], the more modules a network has, the higher the number of negative connections among them. This assumption can partially explain the extremely low PEP values observed for the banana root endosphere networks (0.74 and 0.65% of positive edges for mother plants and suckers, respectively), since both of them have very high modularity values (Table 4) considering the low evenness of these communities (Figure 1). Furthermore, these authors claimed that cosmopolitan individuals tend to establish positive connections, which may suggest that the majority of the endophytes present in the communities here studied could be endemic. Negative interactions in this kind of network may be due to a wide range of co-exclusion mechanisms, including direct competition, environmental modification, differential niche adaptation, and toxin production [84,85]. Further research is needed to shed light on this intriguing phenomenon. Another relevant result obtained from mother plants and suckers networks is that two different ASV of the most abundant bacterial genus, *Pseudomonas*, play important roles in both networks either as a connector in mother plants or as module hub in suckers (Figure 4). This fact highlights the apparent crucial role of *Pseudomonas* in the banana root endophytome. 

An applied objective of our study was to identify, characterize, and evaluate the antagonistic effect and biocontrol potential against *Foc* of indigenous inhabitants of the banana root endosphere. The premise that novel, indigenous BCA can be identified in the same ecological niche colonized by the pathogen has been previously tested and confirmed in our previous studies when targeting a different soil-borne, vascular fungal pathogen [65,71]. In the case of antagonists able to develop an endophytic lifestyle, it is assumed that they have the advantage of being adapted to the plant interior and thus able to favorably compete with the pathogen for infection sites, space, and nutrients [86]. We have generated a large collection of bacterial and fungal endophytes from healthy banana roots. Several interesting conclusions were reached when characterizing this culture collection. Firstly, the number of genera identified was rather low in agreement with the low diversity observed from high-throughput sequencing. Secondly, in addition to *Foc* antagonists identified in the collection, the presence of potential human, plant, and insect pathogens was also detected in the banana root endosphere, which is in agreement with previous studies [1,75]. Thirdly, the phenotyping of Canarian banana root endophytes revealed the presence of activities traditionally associated with PGP and biocontrol for most of the isolates analyzed. 

Despite the fact that many of the culturable banana endophytes showed antagonism against *Foc* STR4 and TR4, the final selection (based on a combination of geographical, molecular, and phenotypic criteria) to be tested *in planta* only showed a slight trend in the reduction of FWB severity for strain *P. chlororaphis* IAS-B-364. One possible explanation for this result could be the cultivar chosen for the biocontrol experiments (Grand Nain), which is different from the original source (Dwarf Cavendish) of the endophytes tested, what may suggest a genotype-dependent effect. In fact, only our reference strain *P. simiae* PICF7 showed significant biocontrol, although in just one of the experiments carried out (Figure 6). It is well known that the biocontrol performance of a given BCA, a synthetic community, or compost application may vary among experiments, and that inconsistency in the effectiveness to suppress a pathogen is not rare [87,88,89,90,91]. Moreover, the best in vitro antagonist is not always the best BCA ([71] and references therein). Strain PICF7 has been previously shown as a highly-versatile beneficial bacterium [92,93,94]. However, the fact that a “foreign” bacterium performed better than the native banana root endophytes here selected was somehow unexpected. These outcomes deserve future attention in relation to (i) the possible superior colonization ability of banana roots by PICF7 and (ii) the potential alterations that its introduction might cause in the composition, structure, and co-occurrence networks of the indigenous banana endophytic communities, which eventually may lead to better biocontrol performance. Small or transient changes in the plants microbiome have been reported after the application of a BCA in crops threatened by a pathogen [95,96,97,98,99]. Whether disturbances in the native communities upon the introduction of “foreign” microbe(s) may cause a “collateral” biocontrol effect mediated by indigenous microbes is not known.

## 5. Conclusions

Taking into account all the results, our hypothesis that root endosphere microbial communities from mother plants and suckers were similar must be partially rejected. While roots of banana plants at different phenological stages showed a comparable composition in their endophytic microbial communities, they only share a small core microbiome, and their co-occurrence network topologies were very different. Therefore, the banana phenological stage determines, at least to some extent, the recruitment and building up of the root endophytome. In contrast, differences in geographic origin (islands) and soil physical–chemical properties did not play relevant roles to shape the composition and structure of the banana root endophytic microbial communities. Moreover, the hypothesis that the banana root endosphere is a good source of microbes with agro-biotechnological potential can be accepted. However, the biocontrol of FWB by the selected candidates here examined could not be confirmed, at least under our experimental conditions. Nevertheless, additional indigenous bacterial and fungal isolates present in the collection are candidates yet to be tested. The effectiveness of the strain PICF7 as BCA toward FWB, alone or in combination with native banana endophytes, deserves to be further investigated under diverse environmental scenarios, including field conditions. The knowledge gathered by combining culturable and non-culturable approaches, as implemented in our study, will pave the way for designing functionally reliable and more efficient synthetic communities to improve banana fitness and assist in FWB management strategies.

## Figures and Tables

**Table 1:** Geographic and crop management information of the banana farms surveyed in this study from Tenerife, La Palma, and La Gomera islands.

Farm Code	Farm Name	Island	Latitude (N)	Longitude (W)	Altitude (m.a.s.l.)	Management ^1^	Fusarium Wilt Incidence (%)
F01	Siverio	Tenerife	28°10′06′′	16°26′13′′	37	IPM	<5
F02	Temaso	Tenerife	28°09′00′′	16°47′36′′	84	IPM	<5
F03	Servicios Agrícolas Abdul	Tenerife	28°11′02′′	16°47′01′′	326	IPM	25
F04	Malpaís-Colpon Agrícola	Tenerife	28°22′36′′	16°44′08′′	22	IPM	<5
F05 *	Fco Pacheco, Arico	Tenerife	28°10′57′′	16°27′00′′	189	IPM	<5
F06 *	La Caldera, Adeje	Tenerife	28°04′34′′	16°43′08′′	113	IPM	<5
F07 *	Siso, Fuencaliente	La Palma	28°28′36′′	17°51′58′′	29	IPM	<5
F08 *	Ortiz, Tijarafe/	La Palma	28°41′47′′	17°57′51′′	300	IPM	35
F09 **	Escuela Capataces	Tenerife	28°29′46′′	16°25′15′′	299	Organic	0
F10 *	Hermigua/	La Gomera	28°10′11′′	17°11′53′′	246	Conventional	<5
F11 *	David, San Sebastián/	La Gomera	28°06′31′′	17°08′32′′	82	IPM	<5

* Farms selected to study the root endophytome by high-throughput sequencing. ** Farm sampled in two consecutive years and used as control. ^1^ IPM: Integrated Pest Management. Farms complying with regulations for the integrated production of bananas from the Canary Islands as well as the private regulations of Globalgap. Organic: Farms complying with the European Regulation for organic farming (Reg CE 834/2007 and further rules that developed it). Synthetic phytosanitary products and inorganic fertilizers are not allowed (fertilization is done by organic materials, compost or some natural substances, such as phosphate rocks, calcium from seashells, etc.). Conventional: Farms complying with basic agricultural regulations. Only authorized banana products are used, and fertilization must be based on a soil analysis. m.a.s.l.: meters above sea level.

**Table 2:** *p*-values of richness (Observed ASV) and Shannon α-diversity indices for the different comparisons analyzed.

		Richness (Observed ASV)		Shannon Index	
Dataset	Comparison	Bacteria	Fungi	Bacteria	Fungi
Tenerife	Farms	0.090	0.207	0.538	0.098
La Palma	Farms	0.822	0.272	0.820	***0.034*** ***(F07* vs. *F08)***
La Gomera	Farms	0.493	0.700	0.608	***0.034***
Tenerife mothers	Farms	0.223	0.316	0.509	***0.049*** ***(F05* vs. *F06, F06* vs. *F09)***
Tenerife suckers	Farms	0.225	0.304	0.904	0.908
La Palma mothers	Farms	0.107	0.256	0.190	0.117
La Palma suckers	Farms	0.538	0.256	0.665	0.075
La Gomera mothers	Farms	0.740	0.304	0.904	***0.001*** ***(F10* vs. *F11)***
La Gomera suckers	Farms	0.538	0.601	0.190	0.117
Mothers	Farms	0.445	0.227	0.603	***0.011*** ***(F10* vs. *F11, F06* vs. *F10)***
Suckers	Farms	0.232	0.612	0.862	0.699
F09/F09 *	Farm	0.058	0.351	0.211	0.592
F09/F09 mothers *	Farm	0.878	0.949	0.354	0.248
F09/F09 suckers *	Farm	0.180	0.305	0.230	0.638
Mothers	Islands	0.418	0.612	0.466	0.699
Suckers	Islands	0.418	0.357	0.466	0.807
Tenerife	Plants	***0.025*** ***(Mothers* vs. *Suckers)***	0.822	***0.021*** ***(Mothers* vs. *Suckers)***	0.956
La Palma	Plants	0.228	0.426	0.085	0.069
La Gomera	Plants	0.061	0.705	***0.013*** ***(Mothers* vs. *Suckers)***	0.069
F09/F09*	Plants	0.204	0.535	0.118	0.420

Farms (comparison among farms), Plants (comparison between mother plants and suckers), and Islands (comparison among Tenerife, La Palma, and La Gomera). Significant *p*-values and its correspondent significant comparisons are shown in bold and italics. * F09/F09: comparison of results from farm F09 obtained in two consecutive years.

**Table 3:** *p*-values of PERMANOVA analysis of quantitative β-diversity index for the different comparisons analyzed: among Farms, Plants (mother plants and suckers), and Islands (Tenerife, La Palma, and La Gomera).

		Weighted Unifrac	Bray–Curtis
Dataset	Comparison	Bacteria	Fungi
Tenerife	Farms	***0.025*** ***(F05* vs. *F06)*** ***(F09* vs. *F05)*** ***(F09* vs. *F06)***	***0.001*** ***(F05* vs. *F06)*** ***(F05* vs. *F09)*** ***(F06* vs. *F09)***
La Palma	Farms	0.385	0.160
La Gomera	Farms	0.968	***0.026*** ***(F10* vs. *F11)***
Tenerife mothers	Farms	0.116	***0.027*** ***(F05* vs. *F06)*** ***(F05* vs. *F09)*** ***(F06* vs. *F09)***
Tenerife suckers	Farms	0.320	0.191
La Palma mothers	Farms	0.338	***0.011*** ***(F07* vs. *F08)***
La Palma suckers	Farms	0.658	0.572
La Gomera mothers	Farms	0.320	0.122
La Gomera suckers	Farms	0.338	***0.049*** ***(F10* vs. *F11)***
Mothers	Farms	***0.009*** ***(F05* vs. *F11)*** ***(F07* vs. *F05)*** ***(F07* vs. *F06)*** ***(F09* vs. *F07)*** ***(F10* vs. *F05)*** ***(F10* vs. *F06)*** ***(F10* vs. *F07)***	***0.002*** ***(F05* vs. *F11)*** ***(F07* vs. *F10)***
Suckers	Farms	0.703	0.071
F09/F09 *	Farm	0.211	***0.002*** ***(F09* vs. *F09)***
F09/F09 mothers *	Farm	0.485	0.060
F09/F09 suckers *	Farm	0.265	***0.018*** ***(F09* vs. *F09)***
Mothers	Islands	0.700	***0.050*** ***(Tenerife*** **vs. *La Palma)*** ***(Tenerife*** **vs. *La Gomera)***
Suckers	Islands	0.561	***0.001*** ***(Tenerife*** **vs. *La Palma)*** ***(Tenerife*** **vs. *La Gomera)*** ***(La Palma*** **vs. *La Gomera)***
Tenerife	Plants	0.315	0.113
La Palma	Plants	0.170	0.811
La Gomera	Plants	0.350	***0.029*** ***(Mothers* vs. *Suckers)***
F09/F09 *	Plants	0.328	0.390

Significant *p*-values and its correspondent significant comparisons are shown in bold and italics. * F09/F09: comparison of results from farm F09 obtained in two consecutive years.

**Table 4:** Main topological properties of co-occurrence networks of mother plants and suckers.

Community	No. Of Original ASV	Similarity Threshold (St)	Total Nodes	Total Links	Percentage of Positive Edges (PEP)	R^2^ of Power- Law	Average Degree (avgK)	Avg Clustering Coefficient (avgCC)	Avg Path Distance (GD)	Modularity (M)
Mothers	323	0.87	127	136	0.74%	0.893	2.142	***0.004***	***6.342***	***0.757*** (17)
Suckers	245	0.88	131	153	0.65%	0.878	2.336	***0.078***	***6.580***	***0.816*** (15)

Significant coefficients (*p*-values < 0.02) between suckers and mothers are shown in bold and italics. Numbers in brackets indicate modules that make up each network. ASV, Amplicon Sequence Variants; GD, Geodesic distance.

**Table 5:** Evaluation of phenotypes traditionally associated with biocontrol and/or plant growth promotion. Assessment was performed in a collection of 122 preselected banana root endophytes that showed in vitro antagonism against *Fusarium oxysporum* f. sp. *cubense*.

*Phenotype*	*Number of Isolates*	*Percentage of Isolates (%)*
Catalase	118	96.7
Phytase	90	73.8
Siderophores	88	72.1
Protease	83	68
Phosphatase	75	61.5
β-glucosydase	49	40.2
HCN	49	40.2
Butanediol	26	21.3
Amylase	16	13.1
Xylanase	9	7.4

**Table 6:** Selected endophytic *Pseudomonas* spp. based on the number of plant growth promotion traits and their ability to in vitro antagonize *Fusarium oxysporum* f. sp. *cubense.*

ISOLATE	Molecular ID *	Island/Farm	In Vitro Antagonism against *Foc*		N° of PGP and Biocontrol Activities
			TR4	STR4	
IAS-B-197	*P. chlororaphis*	Tenerife/Farm 011	Yes	Yes	6
**IAS-B-364**	***P. chlororaphis***	Tenerife/Farm F09	Yes	Yes	7
IAS-B-481	*P. chlororaphis*	Tenerife/Farm F02	Yes	Yes	7
**IAS-B-793**	***Pseudomonas protegens***	Tenerife/Farm F04	Yes	Yes	6
IAS-B-931	*P. chlororaphis* subsp. *aurantiaca*	La Palma/Farm F07	Yes	Yes	6
**IAS-B-944**	***P. chlororaphis* subp. *aureofaciens***	La Palma/Farm F07	Yes	Yes	5
IAS-B-966	*P. chlororaphis* subsp. *piscium*	La Palma/Farm F08	Yes	Yes	5
IAS-B-1013	*P. chlororaphis*	Tenerife/Farm F09	Yes	Yes	5
IAS-B-1054	*P. chlororaphis aureofaciens*	Tenerife/Farm F09	Yes	Yes	5

* Based on DNA sequencing of the *gyrB* and *16S rRNA* genes. PGP: Plant Growth Promotion. Isolates in bold type were selected for biocontrol experiments.

## Data Availability

The datasets generated and analyzed during the current study are available in the NCBI Sequence Read Archive (SRA) under the BioProject number PRJNA575333, and under accession numbers MT445188-MT445196 and MT465269-MT465277. All microorganisms isolated in this study are deposited in the culture collection of the Institute for Sustainable Agriculture (CSIC), Córdoba, Spain. In accordance with Article 17, paragraph 2, of the Nagoya Protocol on Access and Benefit-Sharing Clearing-House (ABS-CH), access permit to collect the genetic resources here described were issued by the ‘Dirección General de Biodiversidad y Calidad Ambiental del Ministerio para la Transición Ecológica’, Spain, with the Prior Informed Consent (PIC) of the ‘Dirección General de Protección de la Naturaleza del Gobierno de Canarias’, Spain. Reference number of the permit: ESNC34. ABS-CH Unique Identifier (UID): ABSCH-IRCC-ES-242814-1.

## Supplementary Materials

The following information is available online at https://www.mdpi.com/2309-608X/7/3/194/s1, Figure S1: Geolocation of banana farms in Canary Islands surveyed in this study. Image adapted from Google Earth (https://earth.google.com/ (accessed on 23 January 2021)), Figure S2: Schematic representation of sampling points to collect roots of banana mother plants and suckers, Figure S3: Box plot of α-diversity indices: observed ASV, Chao1, Shannon, and InvSimpson for the bacterial communities of La Palma (a), Tenerife (b), and La Gomera (c) islands, comparing mother plants and suckers. Significant comparisons are marked with an asterisk (*p*-value < 0.05), Figure S4: Box plot of the α-diversity indices: observed ASV, Chao1, Shannon, and InvSimpson for the fungal communities of mother plants in La Palma (a), Tenerife (b), and La Gomera (c) islands, comparing farms. Significant comparisons are marked with an asterisk (*p*-value < 0.05), Figure S5: Genera showing significant changes in the bacterial community of the ‘Dwarf Cavendish’ root endosphere at Canary Islands. Blue: Mother plants. Green: suckers, Figure S6: Genera showing significant changes in the bacterial community of the ‘Dwarf Cavendish’ root endosphere at La Palma Island. Blue: Mother plants. Green: suckers, Figure S7: Genera showing significant changes in the bacterial community of the ‘Dwarf Cavendish’ root endosphere at Tenerife Island. Blue: Mother plants. Green: suckers, Figure S8: Genera showing significant changes in the bacterial community of the ‘Dwarf Cavendish’ root endosphere at La Gomera Island. Blue: Mother plants. Green: suckers, Figure S9: Genera showing significant changes in the fungal community of the ‘Dwarf Cavendish’ root endosphere at Canary Islands. Blue: Mother plants. Green: suckers, Figure S10: Genus showing significant change in fungal community of the ‘Dwarf Cavendish’ root endosphere at Canary Islands of mother plants, Table S1: Bacterial genera relative abundance in three different Canary Islands (Tenerife, La Palma, and La Gomera), farms/island, plants, mother plants and suckers, and mother plants and suckers/island, Table S2: Fungal genera relative abundance in three different Canary Islands (Tenerife, La Palma, and La Gomera), farms/island, plants, mother plants and suckers, and mother plants and suckers/island, Table S3: Core bacteriome of banana roots of cv. Dwarf Cavendish at Canary Islands. The shared core bacteriome is shown with colored cells. Genera in bold and red color showed significant differences between mother plants and suckers among farms and islands, respectively, Table S4: Core mycobiome of banana roots of cv. Dwarf Cavendish at Canary Islands. The shared core mycobiome is shown with colored cells. Genera in bold and red color showed significant differences between mother plants and suckers among farms and islands, respectively, Table S5: Physical–chemical properties of soils in each surveyed banana farm from Canary Islands. Farms F05, F06, and F09 are located in Tenerife, F07 and F08 are located in La Palma, and F10 and F11 are located in La Gomera, Table S6: Molecular identification of 122 selected banana root endophytes showing antagonism against *Foc* R4 (see Experimental procedures for details), Table S7: Relative inhibition indices of nine selected banana root endophytes against *Foc* STR4 and TR4, Table S8: Phenotypes traditionally associated with biocontrol/plant growth promotion for the most promising banana root endophytes.
