# Supplementary material for: The Banana Root Endophytome: Differences between Mother Plants and Suckers and Evaluation of Selected Bacteria to Control Fusarium oxysporum f.sp. cubense
Source: J Fungi (Basel). 2021 Mar 9;7(3):194. doi: 10.3390/jof7030194 (PMC8002102; doi:10.3390/jof7030194)
Supplement: Supplementary file 1 [file jof-07-00194-s001.zip › Supplementary Tables/Supplementary Table S5.docx]

**Supplementary Table S5.** Physical-chemical properties of soils in each surveyed banana farm from Canary Islands. Farms F05, F06 and F09 are located in Tenerife, F07 and F08 in La Palma, and F10 and F11 in La Gomera.

|  | **FARMS** | | | | | | | | | | | | | | | |
| --- | --- | --- | --- | --- | --- | --- | --- | --- | --- | --- | --- | --- | --- | --- | --- | --- |
|  | F05 | | F06 | | F07 | | F08 | | F09 | | F10 | | F11 | |  |  |
| **Physical analysis** | |  | |  | |  | |  | |  | |  | |  | |  |
| Texture | | SL | | CL | | SL | | CL | | CL | | CL | | L | |  |
| % Clay | | 12.2 | | 28.1 | | 8.3 | | 31.9 | | 32.0 | | 36.9 | | 23.6 | |  |
| % Silt | | 26.4 | | 33.9 | | 27.8 | | 38.0 | | 43.6 | | 35.2 | | 34.6 | |  |
| % Sand | | 61.4 | | 38.0 | | 63.9 | | 30.1 | | 24.4 | | 27.9 | | 41.8 | |  |
| **Chemical analysis** | |  | |  | |  | |  | |  | |  | |  | |  |
| pH 1:2.5 | | 8.1 | | 8.0 | | 7.5 | | 7.9 | | 6.8 | | 5.6 | | 7.6 | |  |
| C.E. ext.sat. mS/cm 25^o^C | | 17.68 | | 7.91 | | 2.97 | | 2.44 | | 6.66 | | 4.80 | | 7.42 | |  |
| Organic material % | | 8.23 | | 8.6 | | 4.96 | | 9.70 | | 5.90 | | 7.29 | | 3.41 | |  |
| Phosphorus Olsen mg/Kg | | 398 | | 312 | | 150 | | 418 | | 284 | | 224 | | 244 | |  |
| Calcium meq/100g | | 34.5 | | 27.2 | | 24.1 | | 38.2 | | 24.0 | | 32.6 | | 30.6 | |  |
| Magnesium meq/100g | | 14.3 | | 17.8 | | 8.4 | | 14.9 | | 10.7 | | 14.3 | | 15.8 | |  |
| Potasium meq/100g | | 16.2 | | 7.5 | | 3.5 | | 6.1 | | 7.5 | | 3.6 | | 4.8 | |  |
| Sodium meq/100g | | 7.6 | | 4.8 | | 0.6 | | 2 | | 0.7 | | 0.6 | | 1.0 | |  |
| Satured paste % SAT | | 91.3 | | 81.5 | | 56.5 | | 100.8 | | 63.9 | | 102.4 | | 59.3 | |  |
| Satured paste % SAR | | 12.9 | | 9.7 | | 2.1 | | 1.1 | | 1.7 | | 0.8 | | 1.9 | |  |
| **Soluble cations meq/L** | |  | |  | |  | |  | |  | |  | |  | |  |
| Calcium | | 30.9 | | 16.9 | | 24.1 | | 12.8 | | 25.9 | | 24.9 | | 36.1 | |  |
| Magnesium | | 63.4 | | 25.9 | | 8.4 | | 10.3 | | 31.5 | | 21.2 | | 35.8 | |  |
| Sodium | | 88.6 | | 44.8 | | 7.1 | | 3.7 | | 9.2 | | 3.7 | | 11.6 | |  |
| Potasium | | 40.1 | | 7.4 | | 4.9 | | 1.3 | | 11.2 | | 3.6 | | 5.2 | |  |
| %N | | 0.81 | | 0.62 | | 0.34 | | 0.65 | | 0.39 | | 0.61 | | 0.22 | |  |
| C/N | | 6 | | 8 | | 8 | | 9 | | 9 | | 7 | | 9 | |  |
| Micronutrients  EDTA mg/Kg | | Fe, Mn, Cu, Zn | | Fe,Mn, Cu, Zn | | Fe,Mn, Cu, Zn | | Fe,Mn, Cu, Zn | | Fe,Mn, Cu, Zn | | Fe,Mn, Cu, Zn | | Fe,Mn, Cu, Zn | |  |
| pH kCl | | 8 | | 7.5 | | 7 | | 7.4 | | 6.5 | | 5.4 | | 7 | |  |

L: Loam; SL: Sandy-loam; CL: Clay-loam.
