# Supplementary material for: The Banana Root Endophytome: Differences between Mother Plants and Suckers and Evaluation of Selected Bacteria to Control Fusarium oxysporum f.sp. cubense
Source: J Fungi (Basel). 2021 Mar 9;7(3):194. doi: 10.3390/jof7030194 (PMC8002102; doi:10.3390/jof7030194)
Supplement: Supplementary file 1 [file jof-07-00194-s001.zip › Supplementary Tables/Supplementary Table S7.docx]

**Supplementary Table S7**. Relative inhibition index of 9 selected banana root endophytes against *Foc* STR4 and TR4.

| ***Foc* Races** | **STR4** | | **TR4** | |
| --- | --- | --- | --- | --- |
| **Media** | **PDA** | **NA** | **PDA** | **NA** |
| **Strains** | **RII** | | | |
| IAS-B-197 | 0.38±0.02**e** | 0.62±0.06**ab** | 0.27±0.02**f** | 0.44±0.07**d** |
| IAS-B-364 | 0.45±0.02**cde** | 0.50±0.04**bc** | 0.28±0.04**f** | 0.44±0.02**d** |
| IAS-B-481 | 0.38±0.06**e** | 0.49±0.02**bc** | 0.32±0.02**ef** | 0.46±0.03**cd** |
| IAS-B-793 | 0.56±0.03**bc** | 0.45±0.04**c** | 0.40±0.02**de** | 0.44±0.02**d** |
| IAS-B-931 | 0.61±0.05**ab** | 0.65±0.08**a** | 0.55±0.04**a** | 0.57±0.02**ab** |
| IAS-B-944 | 0.70±0.02**a** | 0.72±0.07**a** | 0.60±0.03**a** | 0.63±0.03**ab** |
| IAS-B-966 | 0.41±0.04**de** | 0.60±0.03**ab** | 0.43±0.01**bcd** | 0.56±0.03**abc** |
| IAS-B-1013 | 0.58±0.04**ab** | 0.71±0.03**a** | 0.51±0.03**abc** | 0.64±0.02**ab** |
| IAS-B-1054 | 0.69±0.07**a** | 0.73±0.03**a** | 0.53±0.05**ab** | 0.65±0.05**a** |
| PICF7 | 0.52±0.05**bcd** | 0.64±0.02**a** | 0.42±0.03**cd** | 0.54±0.05**bcd** |

PDA, potato dextrose agar; NA, nutrient agar; RII, relative inhibition index. Means±SD in a column followed by different letters are significantly different according to Welch's protected Tukey HDS test (*P*= 0.05).
