# Supplementary material for: The Banana Root Endophytome: Differences between Mother Plants and Suckers and Evaluation of Selected Bacteria to Control Fusarium oxysporum f.sp. cubense
Source: J Fungi (Basel). 2021 Mar 9;7(3):194. doi: 10.3390/jof7030194 (PMC8002102; doi:10.3390/jof7030194)
Supplement: Supplementary file 1 [file jof-07-00194-s001.zip › Supplementary Tables/Supplementary Table S8.docx]

**Supplementary Table S8.** Phenotypes traditionally associated with biocontrol/ plant growth promotion for the most promising banana root endophytes

| **Strain** | **Butanediol** | | **Catalase** | | **Phytase** | | **HCN** | | **Protease** | | **Siderophores** | | **B-Glucosidase** | | **Phosphatase** | | **Xylanase** | |  |
| --- | --- | --- | --- | --- | --- | --- | --- | --- | --- | --- | --- | --- | --- | --- | --- | --- | --- | --- | --- |
| **IAS-B-197** | | - | | + | | + | | + | | + | | + | | - | | + | | - | |
| **IAS-B-364*** | | - | | + | | + | | + | | + | | + | | - | | + | | - | |
| **IAS-B-481** | | - | | + | | + | | + | | + | | + | | - | | + | | - | |
| **IAS-B-793*** | | - | | + | | + | | + | | + | | + | | - | | + | | - | |
| **IAS-B-931** | | - | | + | | + | | + | | + | | + | | - | | + | | - | |
| **IAS-B-944*** | | - | | + | | + | | + | | + | | + | | - | | - | | - | |
| **IAS-B-966** | | - | | + | | + | | + | | + | | + | | - | | - | | - | |
| **IAS-B-1013** | | - | | + | | + | | + | | + | | + | | - | | - | | - | |
| **IAS-B-1054** | | - | | + | | + | | + | | + | | + | | - | | - | | - | |
| **PICF7** | | - | | + | | + | | - | | + | | + | | - | | + | | ± | |

+, presence of activity; −, absence of activity; ± ambiguous result, * Strains selected for biocontrol experiments.
