# Supplementary material for: The Banana Root Endophytome: Differences between Mother Plants and Suckers and Evaluation of Selected Bacteria to Control Fusarium oxysporum f.sp. cubense
Source: J Fungi (Basel). 2021 Mar 9;7(3):194. doi: 10.3390/jof7030194 (PMC8002102; doi:10.3390/jof7030194)

Figure S1: Geolocation of banana farms in Canary Islands surveyed in this study. Image adapted from Google Earth (https://earth.google.com/).

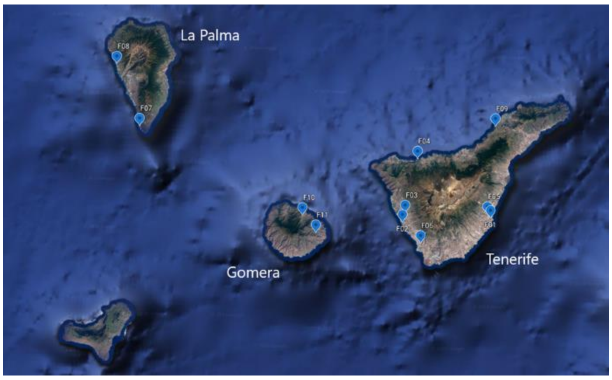

Figure S2: Schematic representation of sampling points to collect roots of banana mother plants and suckers.

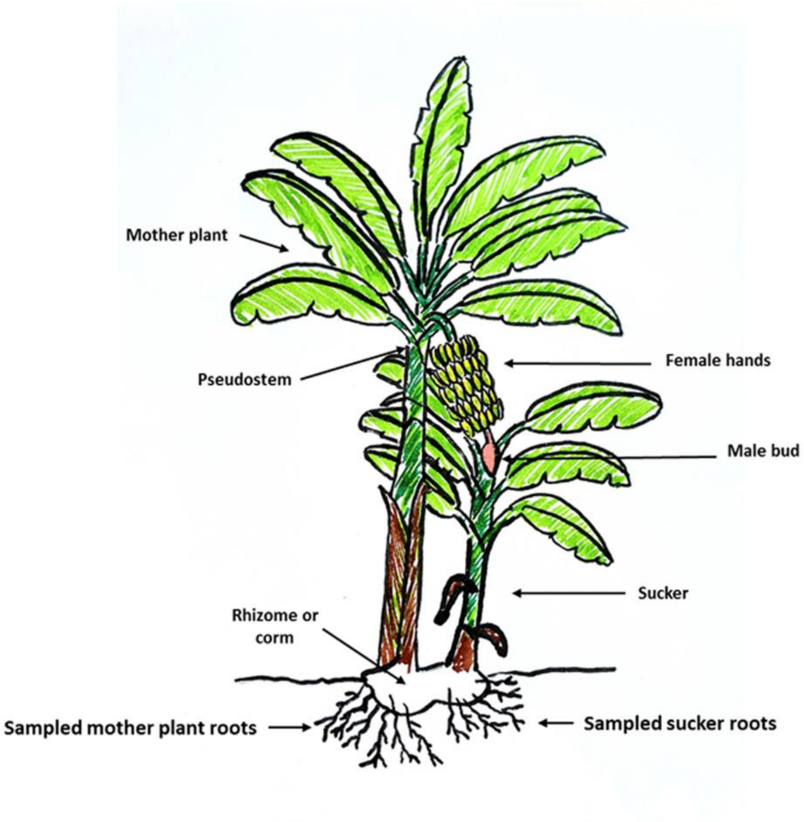

Figure S3: Box plot of α-diversity indices: observed ASV, Chao1, Shannon and InvSimpson for the bacterial communities of La Palma (a), Tenerife (b) and La Gomera (c) islands, comparing mother plants and suckers. Significant comparisons are marked with an asterisk (p-value < 0.05).

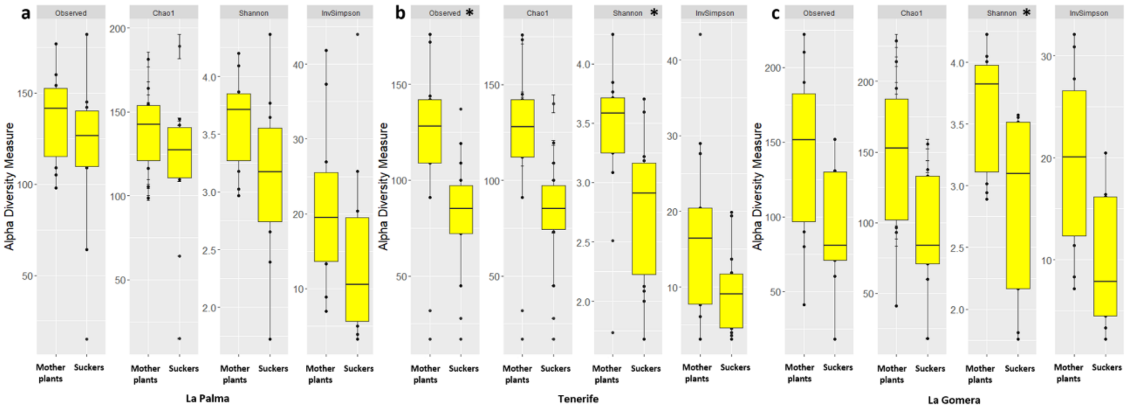

Figure S4: Box plot of the α-diversity indices: observed ASV, Chao1, Shannon and InvSimpson for the fungal communities of mother plants in La Palma (a), Tenerife (b) and La Gomera (c) islands, comparing farms. Significant comparisons are marked with an asterisk (p-value <0.05).

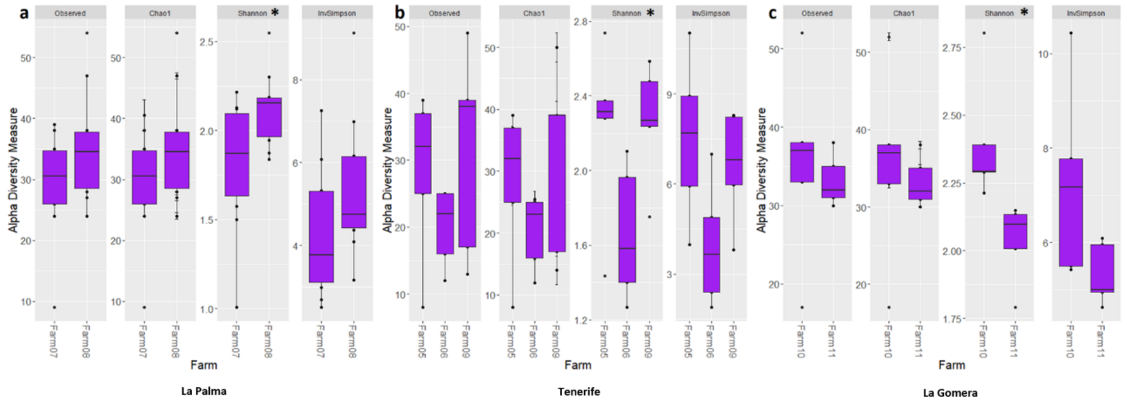

Figure S5: Genera showing significant changes in the bacterial community of the 'Dwarf Cavendish' root endosphere at Canary Islands. Blue: Mother plants. Green: suckers.

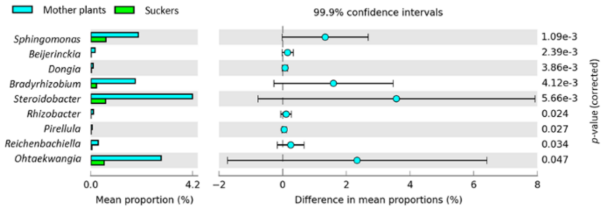

Figure S6: Genera showing significant changes in the bacterial community of the 'Dwarf Cavendish' root endosphere at La Palma Island. Blue: Mother plants. Green: suckers.

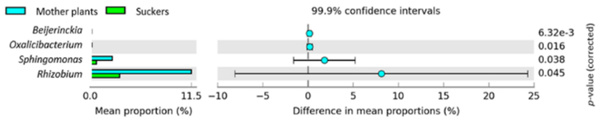

Figure S7: Genera showing significant changes in the bacterial community of the 'Dwarf Cavendish' root endosphere at Tenerife Island. Blue: Mother plants. Green: suckers.

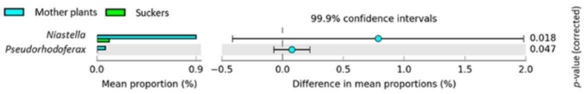

Figure S8: Genera showing significant changes in the bacterial community of the 'Dwarf Cavendish' root endosphere at La Gomera Island. Blue: Mother plants. Green: suckers.

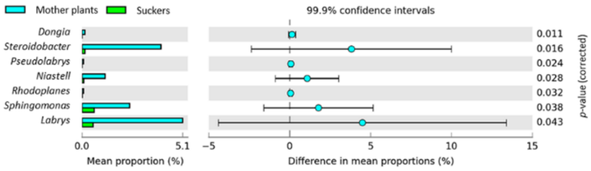

Figure S9: Genera showing significant changes in the fungal community of the 'Dwarf Cavendish' root endosphere at Canary Islands. Blue: Mother plants. Green: suckers.

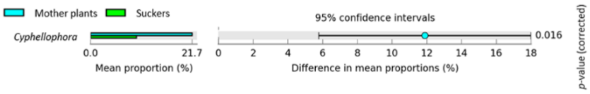

Figure S10: Genus showing significant change in fungal community of the 'Dwarf Cavendish' root endosphere at Canary Islands of mother plants.

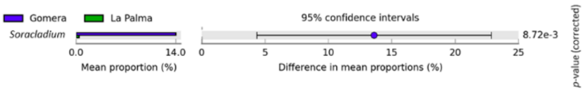

Supplement: Supplementary file 1 [file jof-07-00194-s001.zip › Legend Supplementary figures.docx.pdf]
