# Supplementary figures and images for: The Banana Root Endophytome: Differences between Mother Plants and Suckers and Evaluation of Selected Bacteria to Control Fusarium oxysporum f.sp. cubense
Source: J Fungi (Basel). 2021 Mar 9;7(3):194. doi: 10.3390/jof7030194 (PMC8002102; doi:10.3390/jof7030194)

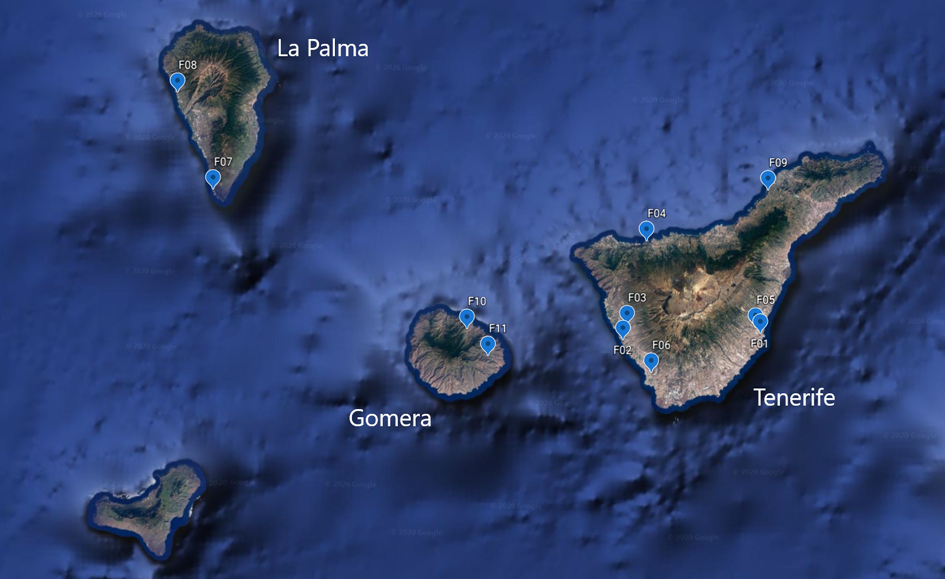

Supplement: Supplementary file 1 [file jof-07-00194-s001.zip › Supplementary Figures/Supplementary Figure S1.tif]

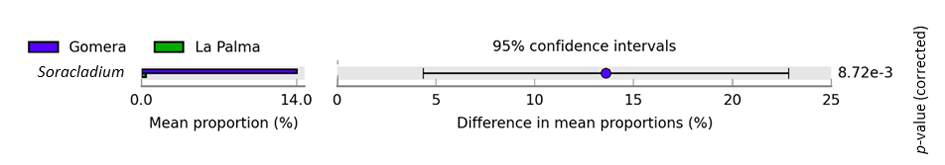

Supplement: Supplementary file 1 [file jof-07-00194-s001.zip › Supplementary Figures/Supplementary Figure S10.tif]

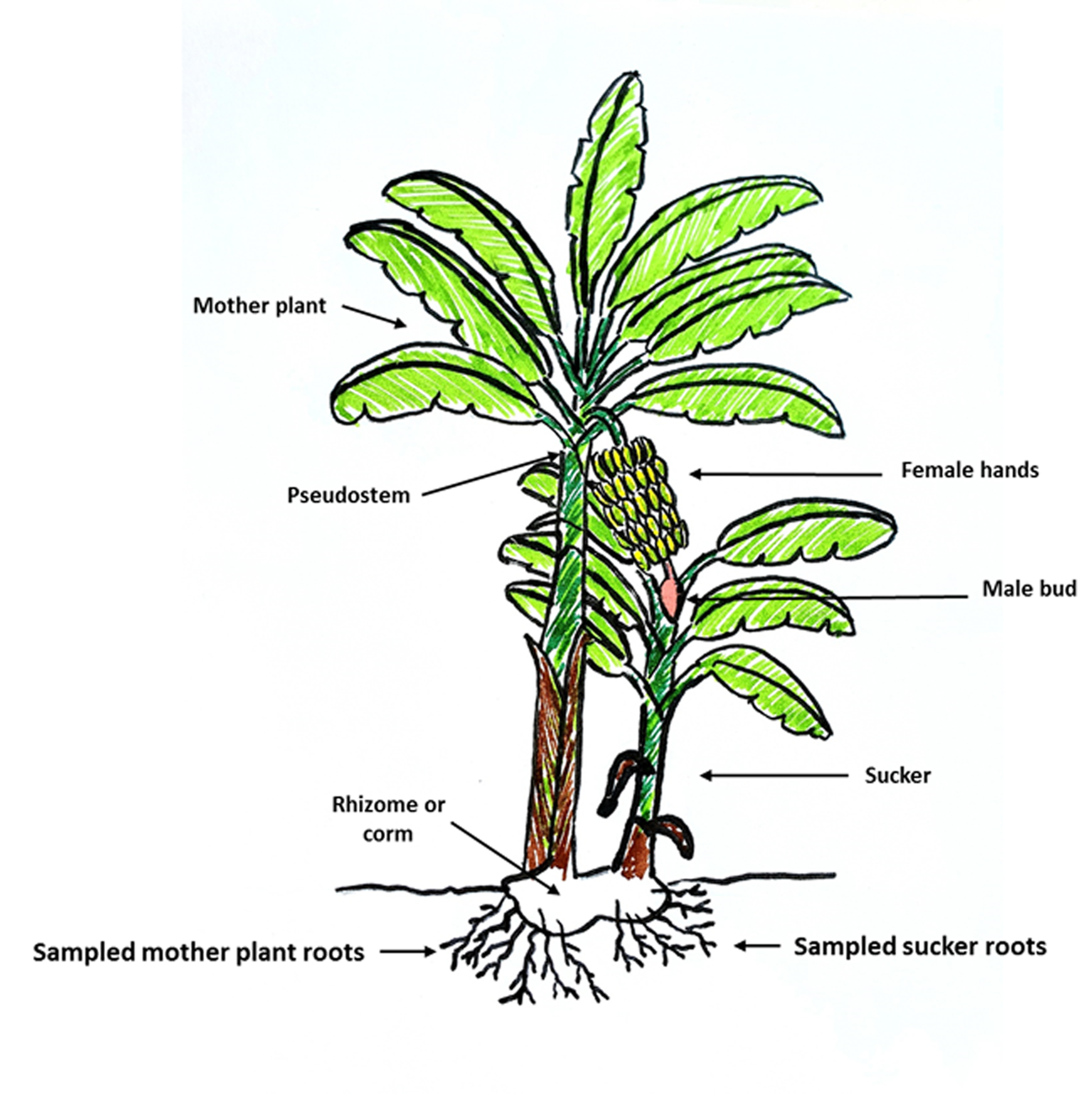

Supplement: Supplementary file 1 [file jof-07-00194-s001.zip › Supplementary Figures/Supplementary Figure S2.tif]

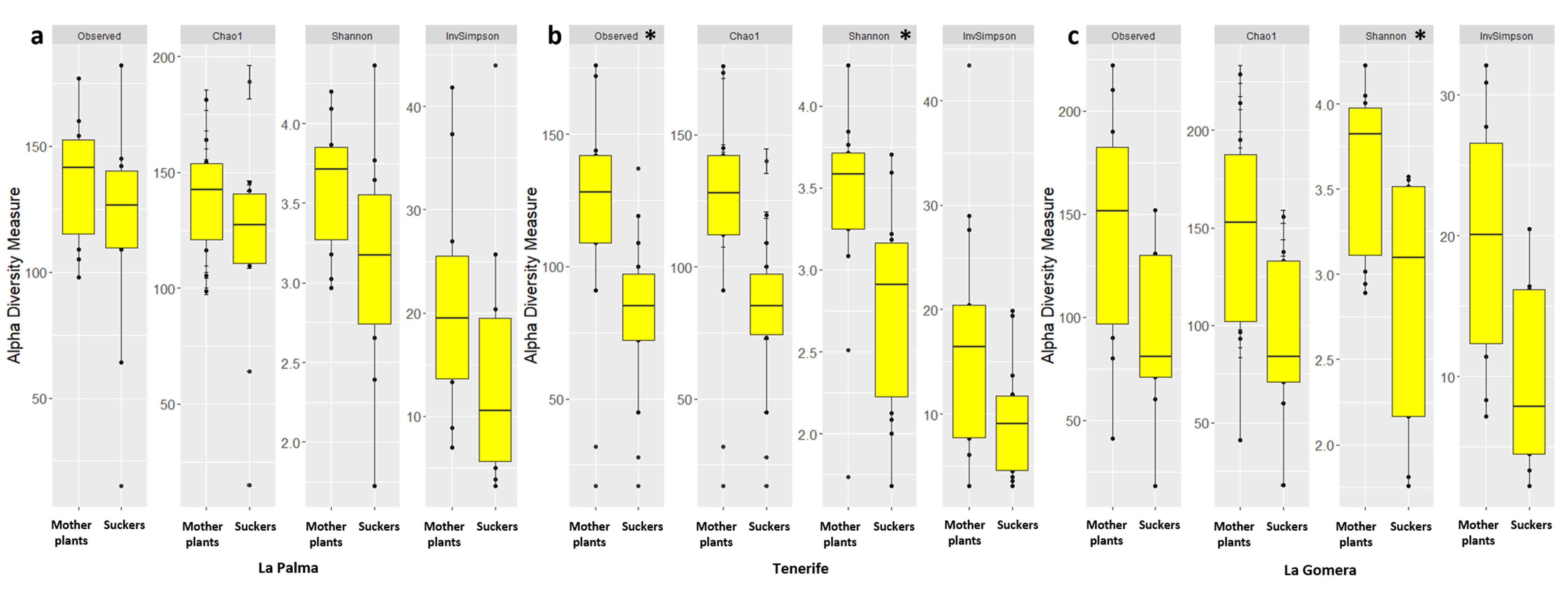

Supplement: Supplementary file 1 [file jof-07-00194-s001.zip › Supplementary Figures/Supplementary Figure S3.tif]

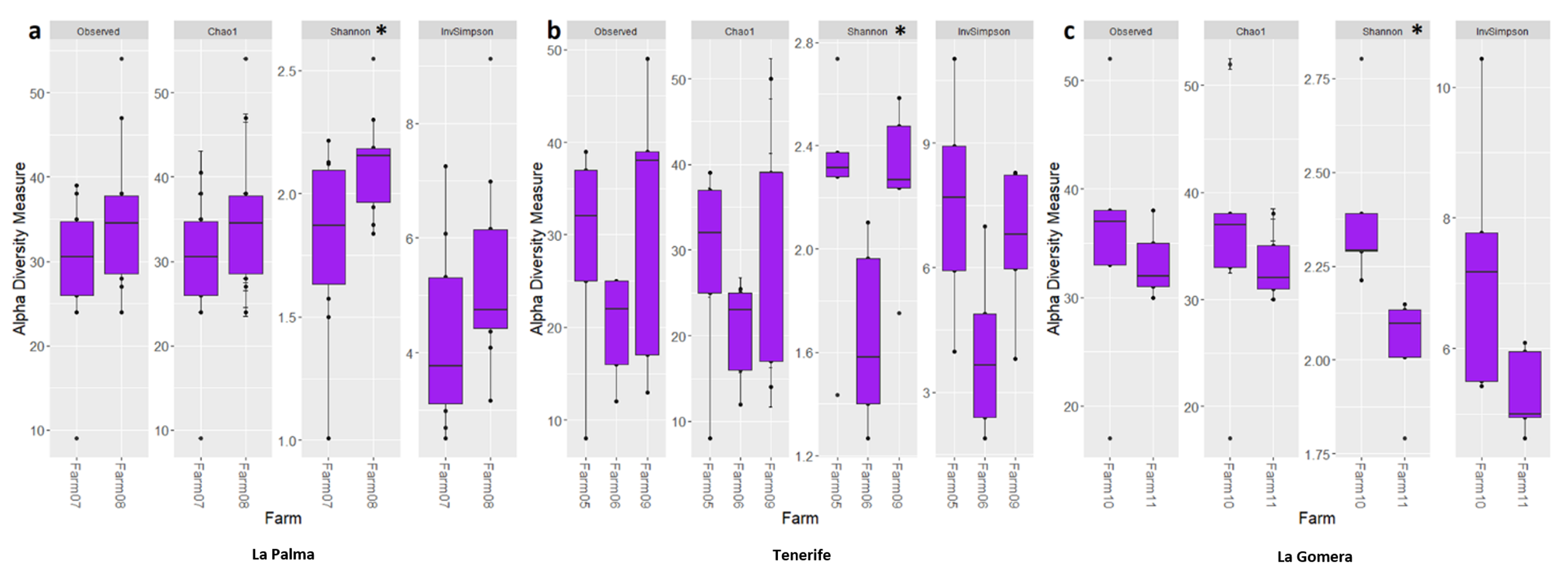

Supplement: Supplementary file 1 [file jof-07-00194-s001.zip › Supplementary Figures/Supplementary Figure S4.tif]

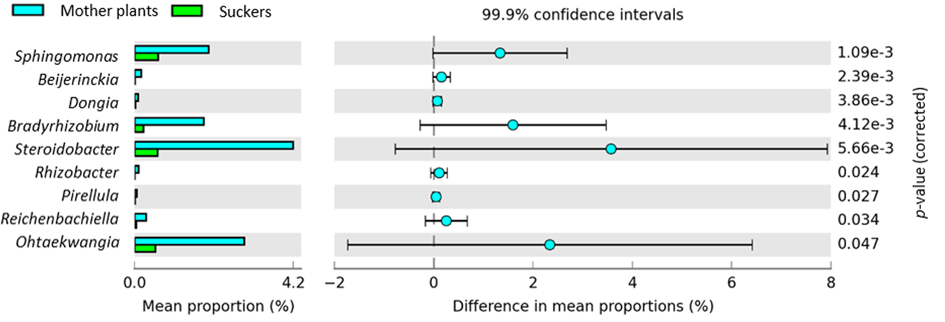

Supplement: Supplementary file 1 [file jof-07-00194-s001.zip › Supplementary Figures/Supplementary Figure S5.tif]

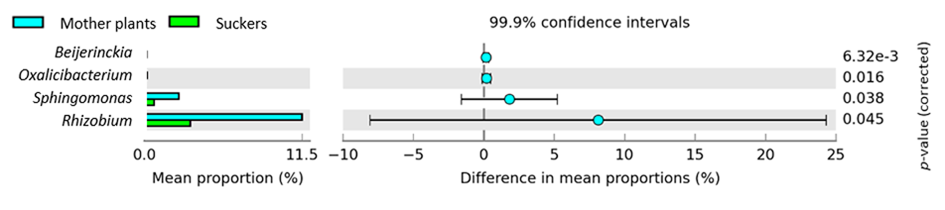

Supplement: Supplementary file 1 [file jof-07-00194-s001.zip › Supplementary Figures/Supplementary Figure S6.tif]

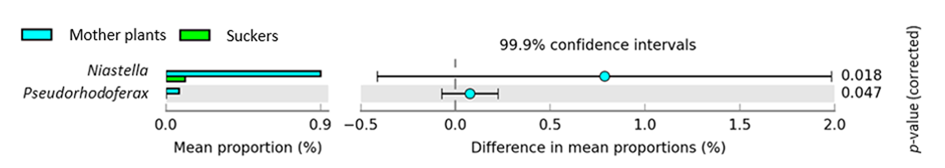

Supplement: Supplementary file 1 [file jof-07-00194-s001.zip › Supplementary Figures/Supplementary Figure S7.tif]

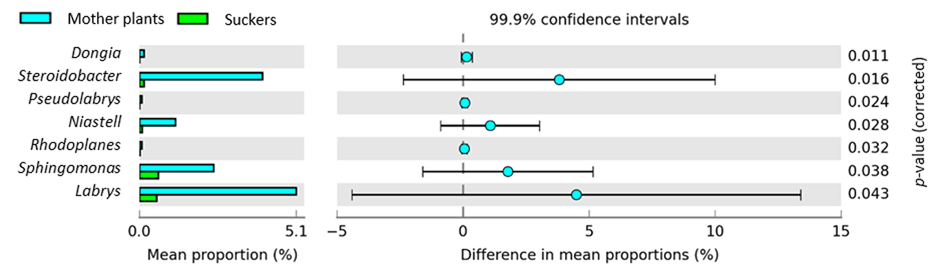

Supplement: Supplementary file 1 [file jof-07-00194-s001.zip › Supplementary Figures/Supplementary Figure S8.tif]

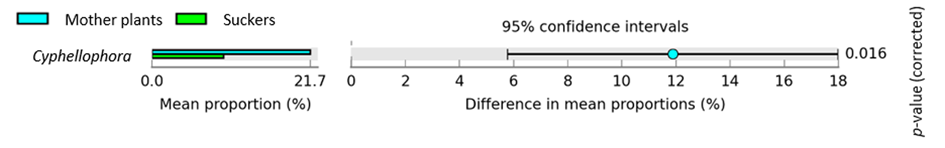

Supplement: Supplementary file 1 [file jof-07-00194-s001.zip › Supplementary Figures/Supplementary Figure S9.tif]
